# Deep learning-driven IoT solution for smart tomato farming

**DOI:** 10.1038/s41598-025-15615-3

**Published:** 2025-08-24

**Authors:** Akshit Saxena, Aayushi Agarwal, Bhavya Nagrath, Carmel Sanjana Jayavanth, Shamita Thulasidoss, S. Maheswari, P. Sasikumar

**Affiliations:** 1 School of Electronics Engineering, Vellore Institute of Technology, Vellore, India; 2 School of Computer Science and Engineering, Vellore Institute of Technology, Chennai, India

**Keywords:** Deep learning, Greenhouse, Tomato production, Precision Agriculture(PA), Internet of things (IoT), Wireless sensor networks (WSN), Electrical and electronic engineering, Environmental impact

## Abstract

The rising food demand and challenges with respect to the climate have made precision agriculture (PA) vital for sustainable crop production. This study presents an IoT-based smart greenhouse platform tailored for tomato farming, integrating environmental sensing and deep learning. The system employs ESP32-based wireless sensors to collect real-time data on soil moisture, temperature, and humidity; this data is transmitted to a cloud dashboard (ThingsBoard) for remote monitoring. A Raspberry Pi equipped with a Pi Camera and a YOLOv8 model classifies tomato ripeness stages—green, half-ripened, and fully ripened—using real greenhouse images. Model optimizations, including quantization, pruning, and TensorRT, improved inference speed by 35% while maintaining 52.8% classification accuracy during our initial stage of the project. Energy profiling revealed daily consumption of 8.91 Wh for the ESP32 sensors and 78 Wh for the Raspberry Pi. This prototype demonstrates real-time monitoring, high model precision, and practical energy insights, paving the way for multi-node scalability and edge AI enhancements. Future work will explore incorporating Edge TPU for faster on-device processing, LoRa for low-power, long-distance data transfer, and automated control of irrigation and ventilation systems to realize a fully autonomous smart greenhouse.

## Introduction

Feeding the world’s growing population is one of the biggest challenges we face today. It is estimated that by the year 2050, the global population will reach around 9.7 billion people, which means food production must increase by about 70% to keep up with demand^1^. However, traditional farming methods often fall short due to limited land, labor shortages, climate change, and the increasing need for sustainable practices^2,3^. Because of this, farmers and scientists are turning to precision agriculture—a modern way of farming that uses smart technologies like the Internet of Things (IoT), Wireless Sensor Networks (WSN), and Artificial Intelligence (AI) to grow crops more efficiently.

This work introduces a smart system designed for greenhouse tomato farming. The system uses sensors, cameras, and deep learning models to monitor environmental conditions and check tomato ripeness automatically. By combining real-time sensor data with image-based detection, our system helps farmers manage their crops better, reduce waste, and improve productivity. The system has been implemented using actual components and tested with real greenhouse images to prove its usefulness.

### The role of IoT and WSN in PA

The Internet of Things (IoT) is a network of smart devices that can collect, share, and act on data. In farming, IoT devices include small sensors that measure things like soil moisture, temperature, humidity, light levels, and more. These devices send the data wirelessly through Wireless Sensor Networks (WSNs) to a central controller, which can make decisions based on the data.

While many existing systems only collect and display this data, our system goes a step further. It uses both sensor values and camera images to help monitor the plants’ condition and take immediate actions, such as turning on a water pump or adjusting the light. This kind of automation helps farmers save time and reduces the chance of human error.

**Fig. 1:**
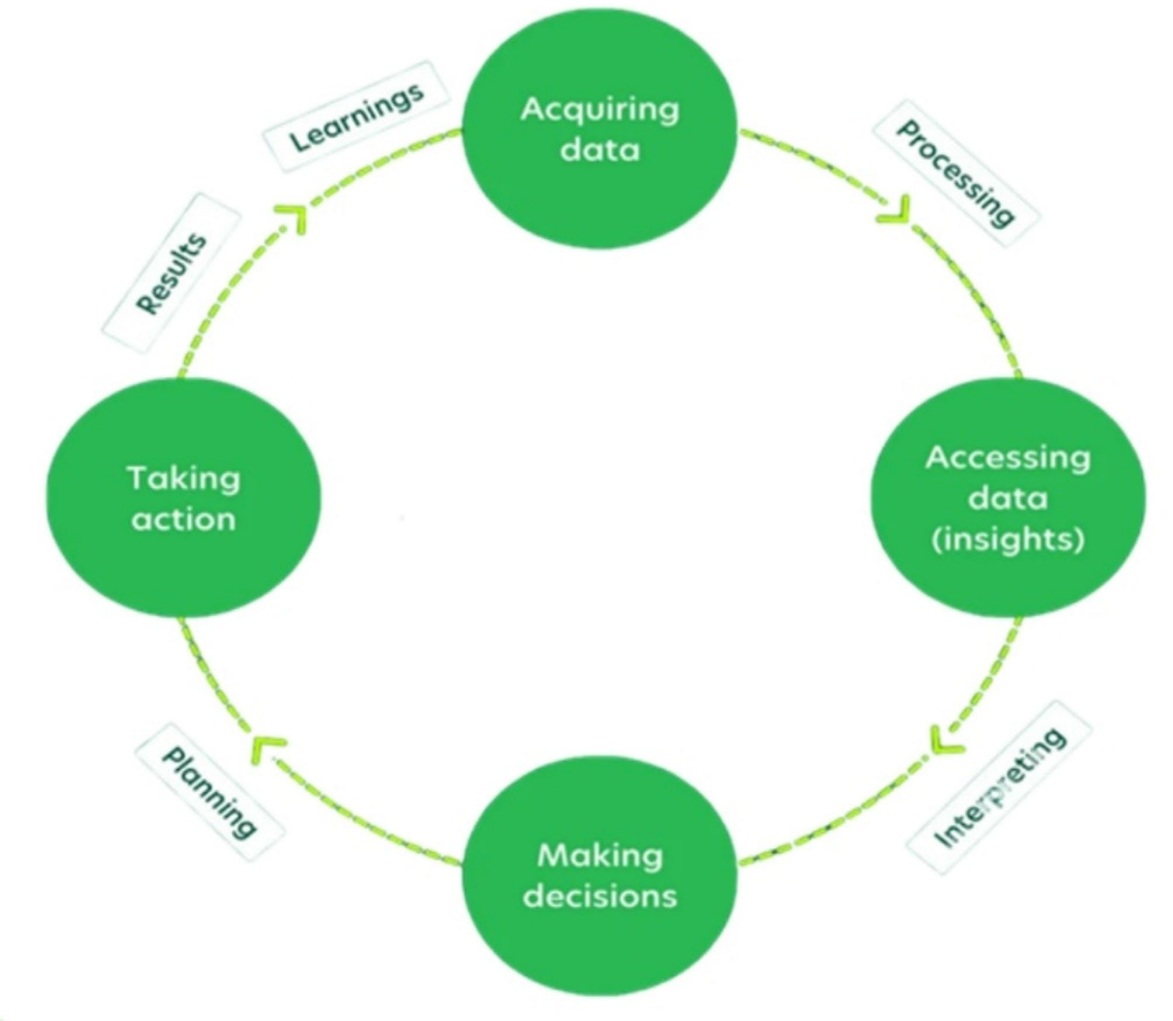
Precision agriculture cycle.

Figure 1 illustrates the precision agriculture (PA) cycle. By continuously monitoring environmental conditions and employing data-driven insights, PA helps farmers optimize resource use, such as water and fertilizers, enhancing crop yields while minimizing environmental impact.

### The focus on tomato crop monitoring

Tomatoes are one of the most grown crops in greenhouses around the world. They are sensitive to environmental changes and need the right temperature, humidity, and light to grow well. They also go through clear ripeness stages from green to yellow to red which makes them suitable for image-based monitoring^8,9^.

Traditionally, farmers determine if tomatoes are ripe by examining them. This method is not always reliable, especially when the farm is large or when different people have different opinions. Using deep learning, particularly models like YOLOv8 (You only look once), tomatoes can be classified accurately into ripeness stages using images. However, many past systems work only in lab settings with perfect lighting and clean backgrounds. Our work focuses on building a system that works in real greenhouse conditions, which include shadows, different light levels, and natural clutter.

### Important IoT uses in the agricultural sector

#### Climate control

Sensors continuously monitor temperature and humidity levels inside greenhouses. The system automatically adjusts ventilation, heating and cooling as needed to maintain stable, optimal growing conditions throughout day and night seasons. This prevents stress on plants and promotes healthy growth.

#### Pest and disease management

IoT devices, such as pheromone traps and camera-based monitoring systems, detect pest infestations and disease outbreaks early. This enables timely interventions, reducing the number of toxic pesticides used and agricultural losses.

#### Irrigation management

Weather forecasts and soil moisture data are used by intelligent irrigation systems to determine the best watering schedules. This minimizes water wastage and makes sure that crops get the appropriate amount of water at the appropriate time.

#### Yield prediction and crop monitoring

IoT devices, combined with remote sensing technologies, provide accurate yield predictions and monitor crop growth and health. Farmers can better manage their crops and plan harvests with the use of this data.

### Sensor technology for real time monitoring

PA utilizes a diverse range of sensors to gather critical data points within greenhouses. These can include:

#### Soil moisture sensors

Continuously monitor soil moisture levels, ensuring optimal water availability for the plants. This prevents overwatering, which can lead to root rot and nutrient leaching, as well as underwatering, which can stress the plants and hinder fruit development.

#### Temperature and humidity sensors

Monitor the temperature and humidity levels within the greenhouse. Maintaining these factors within a specific range is crucial for regulating plant growth, fruit development, and preventing the spread of diseases.

#### Light sensors

Measure the light intensity and spectrum reaching the plants. Tomatoes require specific light levels for optimal photosynthesis. Sensors can trigger adjustments to artificial lighting systems, ensuring consistent light conditions throughout the day, even during periods of low natural light.

#### Imaging sensors

Capture visual data of the plants, enabling the detection of early signs of disease or pest infestation. In this study, imaging sensors were used to collect real-time tomato ripening images from a greenhouse, enhancing the accuracy of the YOLOv8 model.

### Data analysis and decision making

Data from various sensors is sent to a cloud platform or central processing unit. Using advanced data analytics, patterns, trends, and possible problems are identified. Machine learning algorithms, trained in past data, can predict future crop growth and suggest preventive actions. For example, the system may analyze historical temperature and humidity data along with fruit yield to find the best ranges for these factors. This helps farmers adjust greenhouse settings early to maintain ideal conditions for fruit growth and increase yield. The ability to analyze data and make decisions is important for precise tomato production.

Sensor networks in greenhouses collect large amounts of environmental data, but the key is to use this data effectively. Advanced statistical analysis is essential. For example, correlation analysis helps find the best soil moisture levels, which leads to better irrigation and less water waste. Similarly, analyzing past temperature, humidity, and yield data helps predict seasonal trends and adjust greenhouse settings in advance. Machine learning improves this by using historical data to forecast future yield changes based on current and predicted conditions.

This allows farmers to take proactive steps, such as increasing ventilation during a heat wave, to reduce possible yield loss. helpsing risks from pests and diseases also helps farmers prevent outbreaks. Real-time data analysis can send alerts when sensor readings differ from normal values. For example, a sudden rise in temperature or drop in soil moisture can alert farmers to act quickly and protect crops. In summary, data analysis turns raw sensor data into useful information. This gives farmers better knowledge of their crops’ needs, improves resource use, and helps avoid problems before they happen. The result is higher yields, better quality fruit, and a more sustainable and efficient farming system.

### Automatic and improved resource management

Precision agriculture (PA) supports automation in several areas of greenhouse management. For example, irrigation systems can be controlled automatically using real-time soil moisture data. This helps ensure efficient water use and prevents wastage. In the same way, ventilation systems can be adjusted based on temperature and humidity readings to improve energy efficiency.

PA can also be used for lighting control. By using light sensors and timers, the system can follow natural light patterns or provide extra lighting during low-light conditions. This ensures plants receive the right amount of light for photosynthesis, helping improve growth and yield.

In this study, an imaging-based AI approach is also used. This allows for better resource use and helps in planning harvest times more effectively.

### Benefits and future scope

Using Precision Agriculture (PA) for monitoring tomato crops brings several clear benefits. It can help increase yield, improve the quality of the fruit, and reduce the use of water and fertilizers. This leads to lower running costs and less harm to the environment. By using data to guide decisions, farmers can make smarter choices about when to water, ventilate, or harvest, which leads to better overall results.

As technology keeps improving, we can expect to see even more advanced sensors, better data analysis tools, and stronger use of artificial intelligence. These developments will make tomato farming even more accurate and efficient.

This project also shows that it is possible to expand the current setup into a larger IoT system using more sensor nodes. This would help in better performance testing and understanding the system in real farming situations. In the future, mobile apps and user-friendly interfaces can make it easier for farmers to monitor and control greenhouse conditions from anywhere. This gives them more flexibility and supports a new way of farming that is driven by data and smart systems.

## Related work

In recent years, the combination of Internet of Things (IoT) and Wireless Sensor Networks (WSN) has gained significant attention in precision agriculture. These technologies help improve crop yield and resource efficiency by allowing real-time monitoring and control of various environmental factors. Many studies have explored IoT-based systems for crop monitoring, the use of deep learning for agricultural analysis, and cloud platforms for visualizing and managing data. However, most of these works rely on simulated conditions or public datasets, without testing their solutions in real environments.

Our study aims to bridge this gap by using real greenhouse images and deploying a working prototype. This section reviews related works in the field — starting with IoT and WSN systems for environmental monitoring, followed by deep learning applications for crop analysis, and finally cloud-based tools for data processing and visualization. It also highlights how recent efforts have been made to integrate these technologies, and how our approach builds upon them to deliver a more practical, real-world solution.

The increasing demand for food due to rapid population growth has necessitated the adoption of innovative agricultural practices to enhance productivity and sustainability^1^. Innovative robotic system designed for the automated detection of tomato leaf diseases within greenhouses.is proposed in^2^ This system integrates a fuzzy control algorithm for autonomous navigation of the robot with a deep learning model for classifying tomato plant diseases from leaf images. A key contribution of this research is the development of an improved Deep Convolutional Generative Adversarial Network (DCGAN). This DCGAN is utilized to augment the training dataset by generating synthetic images of diseased tomato leaves, significantly enhancing the diversity and size of the dataset. The study meticulously compares the performance of four prominent deep learning architectures (VGG19, Inception-v3, DenseNet-201, and ResNet-152) across nine classes of tomato leaf diseases.

Precision agriculture leverages various technologies like sensors, wireless communication, and cloud computing to monitor and manage agricultural variables with high accuracy. For instance, IoT-driven monitoring of soil moisture, temperature, humidity, and light plays a crucial role in greenhouse environments, particularly in tomato farming, where environmental consistency is vital^3^. Wireless Sensor Networks (WSNs) have become fundamental in facilitating this real-time data collection and control^4^.

The integration of sensor data with automated actuators allows for smart control strategies that help reduce resource wastage, enhance crop yields, and maintain sustainability^5^. A growing number of systems have been proposed that monitor and adjust parameters such as irrigation, ventilation, and lighting within greenhouses^3^. These platforms have proven especially useful in remote areas, where continuous human supervision is impractical^6^.

Recent developments have also incorporated mobile and cloud-based interfaces to allow farmers to remotely visualize data and interact with the system in real time^7^. Cloud services offer scalable storage and real-time analytics that can predict crop behavior, identify stress conditions, and suggest corrective actions^8^.

Edge computing is increasingly being explored to reduce the dependency on constant cloud communication. By using edge devices like Raspberry Pi and ESP32, real-time processing and control can be achieved on-site, enhancing system responsiveness and energy efficiency^9–11^.

Datasets specifically designed for agricultural applications, such as tomato ripeness classification, have supported the training of deep learning models in smart farming^12^. These models can detect ripeness stages or disease symptoms based on multispectral or RGB imagery captured via camera modules integrated into the greenhouse system^13^.

Computer vision and deep learning algorithms have demonstrated impressive results in plant identification, disease detection, and even weed classification, thereby contributing to effective pest control and harvest management^14^. Transfer learning and model optimization techniques further enhance the performance of image classification in real agricultural settings^15^.

There is also considerable research on the use of drone and MAV-based systems for large-scale data acquisition in open-field agriculture, offering scalability and better aerial insights^16^. These tools are often coupled with IoT infrastructures for centralized decision-making and automated actuation^17^.

Literature has pointed to the potential of combining IoT and AI into a unified system that offers both data acquisition and intelligent analysis in real-time^18^. This convergence ensures a higher degree of automation and reduces reliance on traditional farming methods.

Energy consumption, however, remains a critical limitation in WSN-based agricultural systems. Research has focused on optimizing routing protocols and sensor placement to extend system life, especially in off-grid or solar-powered environments^19^. IoT devices deployed for greenhouse automation must, therefore, prioritize energy-efficient communication and computation methods^20^.

Furthermore, several works have demonstrated the efficacy of Arduino-based greenhouses for beginners and educational purposes, offering a low-cost entry into smart agriculture^21^. These setups are not only affordable but also modular, allowing for customization as needed^22^.

Practical applications and field deployments have reinforced the reliability of such IoT systems for greenhouse monitoring and control. Greenhouse tomatoes benefit significantly from such systems due to their sensitivity to environmental variations^23^. Numerous agricultural extension services and online platforms now provide guidance on implementing these technologies at a local level^24^.

In summary, the literature demonstrates that smart greenhouse systems supported by IoT, WSNs, edge computing, and deep learning are shaping the future of agriculture. These interdisciplinary systems enhance environmental control, enable disease detection, and ultimately improve crop quality and yield. The current research builds upon these foundational concepts to develop an integrated smart tomato farming platform, aiming to bridge existing technological gaps while remaining practical and scalable for real-world use.

## Proposed system and specifications

The proposed system integrates IoT, Wireless Sensor Networks (WSN), deep learning algorithms, and cloud computing to create a comprehensive smart agriculture solution. The system is designed to provide real-time monitoring and analysis of crop conditions, enabling farmers to make data-driven decisions to optimize crop yield and resource utilization. This study enhances existing solutions by incorporating real greenhouse images, refining model training through practical data collection, and addressing IoT deployment challenges.

**Fig. 2:**
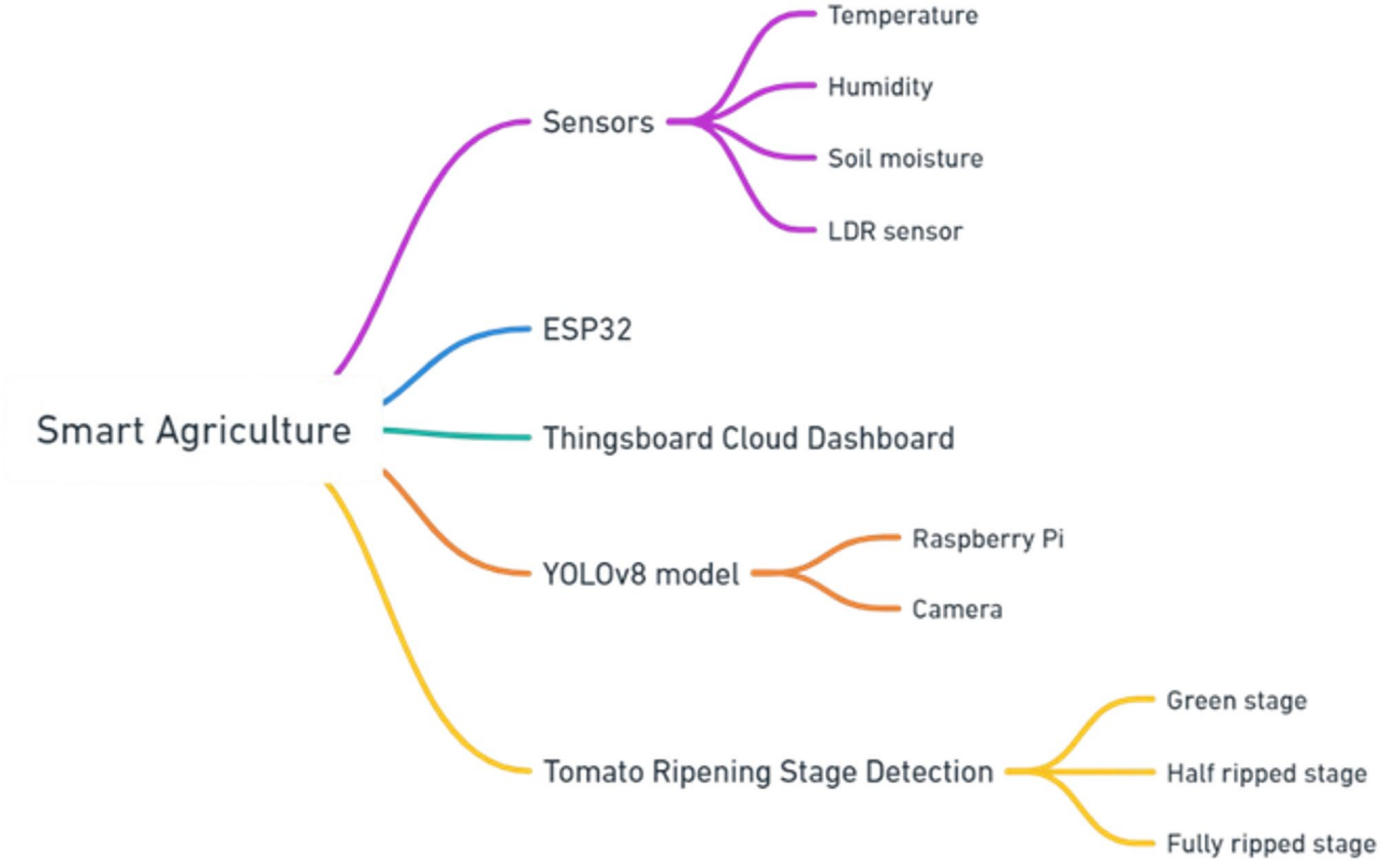
Precision agriculture project structure.

The proposed smart platform as shown in Fig. 2 for monitoring tomato crops in greenhouses comprises three primary components -sensors for temperature, humidity, and soil moisture, an LDR sensor, an ESP32 microcontroller, and a Thingsboard Cloud Dashboard for data visualization. Additionally, it includes a Raspberry Pi with a camera for image capture and a YOLOv8 model for detecting tomato ripening stages (green stage, near-ripe stage, and fully ripe stage), highlighting the integration of IoT and AI in modern farming.

### IoT-based sensor network

Real-time data by deploying a prototype node within a greenhouse to validate real-time data acquisition. The chosen sensors include:

#### Soil moisture sensor

This device determines the moisture level of the soil to allow for targeted irrigation scheduling to maintain ideal moisture levels for optimal root development and nutrient uptake.

#### Temperature and humidity sensor

tracks the humidity and temperature within the greenhouse. This data is crucial for maintaining a comfortable and controlled environment that fosters optimal tomato growth. Excessive temperatures or humidity can lead to stunted growth, disease outbreaks, and reduced fruit quality.

#### Light intensity measurement

LDR sensors can be integrated into the sensor network to measure the amount of light reaching the tomato plants. As light intensity increases, the resistance of the LDR sensor decreases. By measuring this resistance, the system can quantify the light plants are receiving. Utilizing Light Data: The ‘real-time’ data measured by the sensors can be:

#### Monitored

Farmers can remotely view data levels in their greenhouses through the cloud platform (ThingsBoard).

#### Analysed

By observing light trends, they can identify periods of low or excessive data levels.

#### Acted upon

Adjustments can be made to optimize conditions. This might involve using artificial lights to supplement natural light during low-light periods or installing shade cloths to regulate intense sunlight. Sensor-based actions such as opening water shafts or switching heaters could be controlled based on soil moisture and temperature data.

### Multiple units setup

The proposed system has mainly two additional units that are as follows:

#### Storage and transmission unit

An ESP32 microcontroller serves as the central data collection and transmission unit. It collects data from the sensors at predefined intervals (e.g., every minute) and transmits it wirelessly to the cloud platform using Wi-Fi connectivity. The ESP32 offers a cost-effective and efficient solution but is limited by high energy consumption due to continuous Wi-Fi usage. Future work will explore lower-energy alternatives such as LoRa-based transmission.

#### Collection and image processing unit

A Raspberry Pi, a single-board computer, serves as the image storage and processing unit. It is equipped with a pi camera module to capture images of tomatoes at regular intervals (e.g., every hour). The captured images are then processed using the YOLOv8 deep learning model, which is pre-trained to identify and classify the ripening stage of tomatoes (green, half-ripened, and fully ripened). However, future improvements could involve fine-tuning the model with additional greenhouse-specific images to further enhance accuracy.

The YOLOv8 model is chosen for its speed and accuracy in real-time object detection tasks. It efficiently analyses the captured images, identifying tomato objects and assigning bounding boxes around them. Additionally, the model predicts the ripening stage for each detected tomato, providing valuable insights for harvest planning and resource allocation. providing valuable insights for harvest planning and resource allocation. However, this study does not yet address real-time inference efficiency on embedded devices, a potential area for further research.

### Tomato plant environment

To ensure optimal growth and yield of tomato plants, specific environmental conditions and parameters must be maintained within the greenhouse as mentioned in Table 1. These include:

**Table 1:** Physical parameters for tomatoes.

S No.	Parameters	Ideal value
1.	Day temperature	70 °F
2.	Night temperature	60–64 ℉
3.	Day humidity	80–90%
4.	Night humidity	65–75%
5.	pH	Near 7

#### Humidity

Relative humidity levels within the greenhouse should be maintained between 60% and 70%. This range helps to prevent issues such as mould and mildew while ensuring the plants have adequate moisture for optimal transpiration and nutrient uptake.

#### Temperature

Tomato plants thrive in a temperature range of 20 °C to 25 °C (68 °F to 77 °F). Daytime temperatures should ideally be kept around 22 °C to 24 °C (72 °F to 75 °F), while night-time temperatures can be slightly cooler, around 18 °C to 20 °C (64 °F to 68 °F). Extreme temperatures outside this range can negatively impact plant growth and fruit production^6^.

#### Soil moisture

Regular monitoring of soil moisture is crucial. Tomato plants require consistently moist soil, but it should not be waterlogged. Soil moisture levels should be maintained at an adequate level to ensure proper root development and nutrient absorption.

#### Light intensity

Tomato plants need ample light for photosynthesis. In a greenhouse setting, providing around 6 to 8 h of direct sunlight or equivalent artificial lighting per day is ideal. Light intensity should be adjusted based on the growth stage of the plants to promote healthy development and fruiting.

#### Air circulation

Proper air circulation is essential to prevent overheating and humidity buildup. Ensuring good ventilation helps to maintain even temperatures and reduce the risk of fungal diseases. Future system iterations could include IoT-controlled ventilation mechanisms for automation.

### Cloud platform

**Fig. 3:**
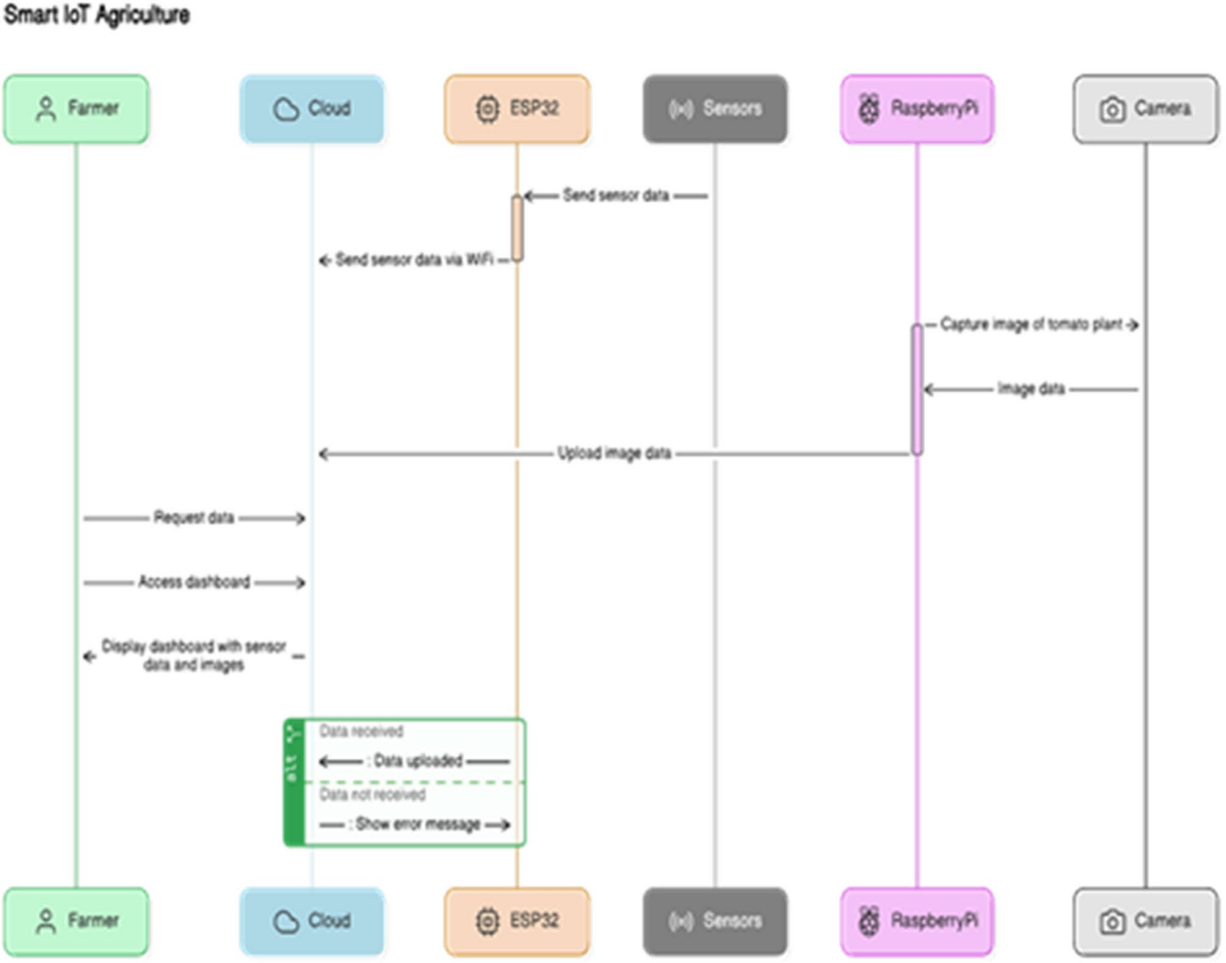
Dataflow of architecture.

ThingsBoard, an open-source IoT platform, serves as the cloud-based platform for analysis, visualization, and data storage. The MQTT (Message Queue Telemetry Transport) protocol, a lightweight messaging protocol designed for Internet of Things applications, is used by the ESP32 to send sensor data to ThingsBoard. Figure 3 depicts the entire IoT architecture.

The Raspberry Pi also uploads the photos taken that show the identified ripening stages to ThingsBoard. ThingsBoard offers an intuitive graphical user interface for remotely accessing recorded photos, historical trends, and sensor data. Users can:

- View real-time and historical data in various formats, including charts and graphs, to gain insights into trends and environmental fluctuations.
- Configure alerts for critical events (e.g., exceeding temperature thresholds or critically low soil moisture levels) to ensure prompt intervention.
- Generate reports for further analysis, enabling farmers to monitor the condition of their crops over time and spot possible problems before they get worse.

## Software implementation: bringing the system to life

**Fig. 4:**
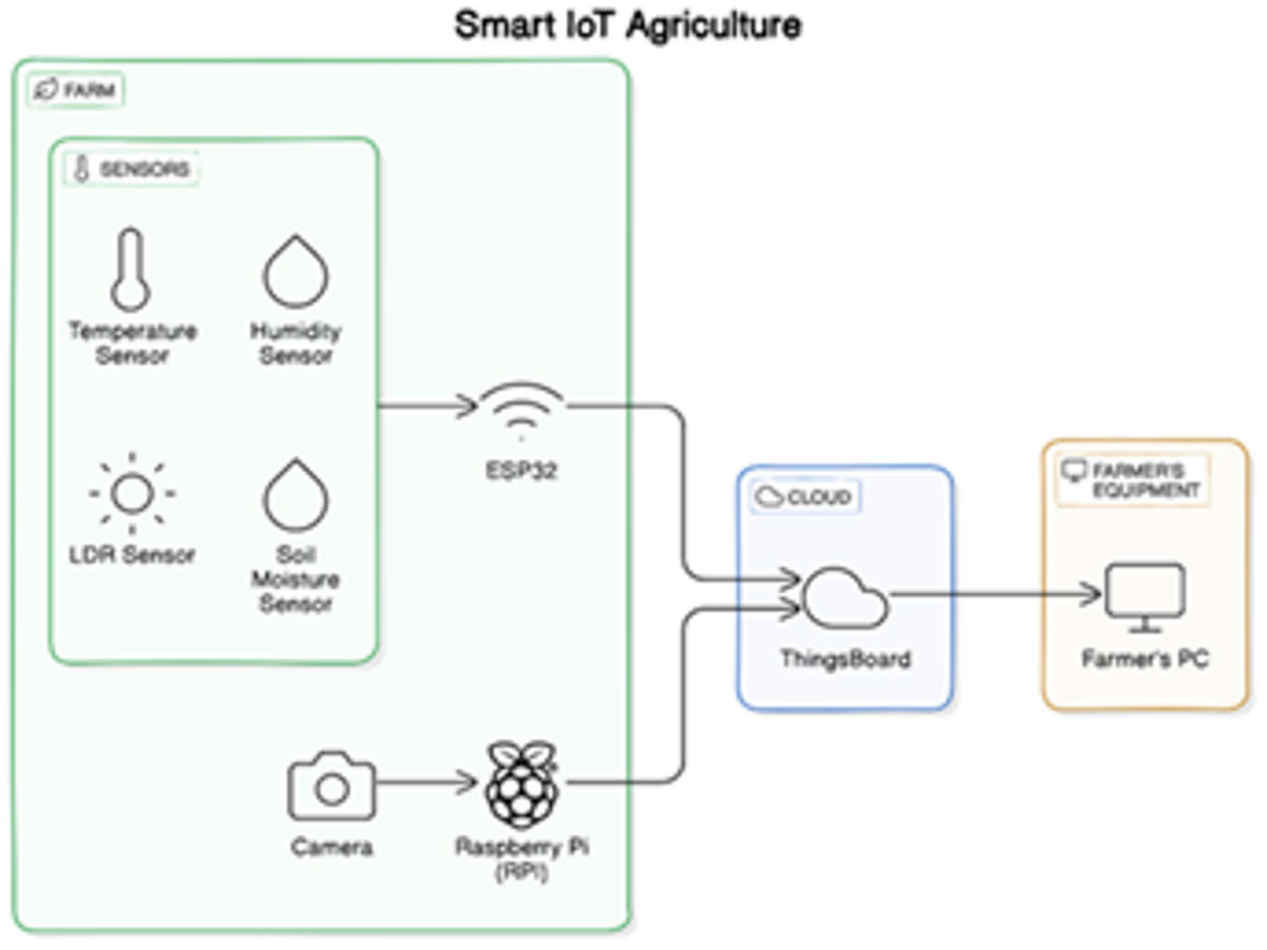
The proposed IoT architecture of smart agriculture.

The software components of the smart platform play an important part in gathering, sending, processing, and displaying data, Fig. 4 shows the flowchart of our proposed Smart IoT Agriculture. Here’s an overview of the software functionalities:

### Sensor data acquisition

ESP32 is programmed using the Arduino IDE (Integrated Development Environment) to collect data from the connected sensors at predefined intervals. Libraries specific to the sensor models are utilized to ensure accurate data readings. The collected data is then formatted and packaged for efficient transmission to the cloud platform.

### Data transmission

Figure 3 below illustrates the data flow in a smart IoT-based agriculture system. The ESP32 microcontroller collects real-time sensor data, such as temperature, humidity, and soil moisture, and transmits it via MQTT to the ThingsBoard Cloud over Wi-Fi. A Raspberry Pi with a camera captures crop images, which are processed locally using models like YOLOv8 to detect ripeness or diseases. The processed insights and live sensor data are displayed on a cloud dashboard or farmer’s PC, enabling informed, real-time decisions. This setup shows how edge devices, cloud platforms, and AI work together to enhance precision farming.

### Image capture and processing

The Raspberry Pi utilizes its operating system (e.g., Raspberry PI OS) and Python programming language to control the camera module for image capture. Libraries like OpenCV (OpenSource Computer Vision Library) are employed for image processing tasks like resizing and format conversion.

### Dataset

“Laboro Tomato is an image dataset of growing tomatoes at different stages of ripening, designed for object detection and instance segmentation tasks. The dataset comprises two subsets of tomatoes separated by size and was gathered at a local farm using two separate cameras with different resolutions and image quality”^5^.

“Each tomato is divided into 2 categories according to size (normal size and cherry tomato) and 3 categories depending on the stage of ripening:

- fully_ripened - completely red color and ready to be harvested. Filled with red color on 90%* or more.
- half_ripened - greenish and needs time to ripen. Filled with red color on 30–89%*.
- Green - completely green/white, sometimes with rare red parts. Filled with red color on 0–30%*.

*All percentages are approximate and differ from case to case.

Dataset includes 804 images with following details^5^:

name: tomato_mixed.

images split: 643 train, 161 test.

cls_num: 6.

cls_names: b_fully_ripened, b_half_ripened, b_green, l_fully_ripened, l_half_ripened, l_green.

total_bboxes: train[7781], test[1,996].

bboxes_per_class:

*Train: b_fully_ripened[348], b_half_ripened[520], b_green[1467].

l_fully_ripened[982], l_half_ripened[797], l_green[3667].

*Test: b_fully_ripened[72], b_half_ripened[116], b_green[387],

l_fully_ripened[269], l_half_ripened[223], l_green[929].

image_resolutions: 3024 × 4032, 3120 × 4160.”

### Model creation

A model based on the YOLOv8n architecture was trained for detecting the ripening stages of tomatoes. The model takes high-resolution images (3024 × 4032 or 3120 × 4160) as a training and validation input. The head, neck, and backbone make up the three primary parts of the YOLOv8 architecture. A convolutional neural network (CNN), which serves as the brains, uses the input images to extract key information. This is followed by the neck, which processes these features through layers such as Feature Pyramid Networks (FPN) or Path Aggregation Networks (PAN). Finally, the head predicts bounding boxes and class probabilities for each detected tomato. The backbone utilizes convolutional layers with kernel sizes of 3 × 3 and activation functions of Rectified Linear Unit (ReLU). MaxPooling layers are applied to each convolutional layer to reduce spatial dimensions, and the data is transformed into a column vector by a global average pooling layer. This vector is linked to a dense layer that utilizes softmax as the activation function and has six output nodes (full, half-ripened, and green, for both regular size and cherry tomatoes).

### Model training

The parameters used to train the Adam optimization algorithm and the YOLOv8n model are shown in Table 2. The loss function of the model was derived by combining binary cross-entropy for classification and mean squared error for bounding box regression, two frequently used functions for object detection tasks. The number of epochs was set to 25 to ensure adequate training time for convergence.

**Table 2:** Parameters used for the training of the model^4^.

Sl. No.	Parameter	Value
1.	‘Batch size’	8
2.	‘Number of epochs’	25
3.	‘Step per epochs’	80
4.	‘Validation steps’	20
5.	‘Image Size’	640
6.	‘Optimization algorithm’	Adam
7.	‘Loss function’	Mean squared error (bbox), Binary cross-entropy (class)

### YOLOv8n model deployment

The YOLOv8 deep learning model is trained based on the dataset and optimized for deployment on the Raspberry Pi. To accommodate IoT-constrained devices with limited computational power, optimization techniques such as model quantization, pruning, and TensorRT acceleration were applied.

- Quantization: Reduces the precision of weights from 32-bit floating point to 8-bit integers, decreasing model size and improving inference speed.
- Pruning: Removes less significant neural connections, reducing computational complexity while maintaining accuracy.
- TensorRT Acceleration: Converts the model into an optimized inference engine for ARM-based hardware.
- Performance Changes: After optimization, the model demonstrated a 35% reduction in inference time while maintaining an accuracy of 50.8% in classifying ripening stages. Compared to the original model, RAM usage decreased by 40%, and power consumption was optimized to ensure sustainable real-time execution on the Raspberry Pi.

Once deployed, the optimized model analyzes the captured images, detecting tomatoes and predicting their ripening stages with improved efficiency. Future work will focus on further reducing latency by exploring Edge TPU-based acceleration for real-time applications.

#### Why YOLOv8

The selection of YOLOv8n for tomato ripeness detection is based on its superior balance of accuracy, speed, and resource efficiency, making it ideal for deployment on edge devices like Raspberry Pi in real-time agricultural scenarios.

- Lightweight and Edge-Optimized: YOLOv8n is significantly more efficient than previous versions and other detection models, making it suitable for real-time inference on Raspberry Pi—a key requirement for our system.
- High Accuracy with Small Objects: With an anchor-free architecture and improved bounding box regression, YOLOv8n delivers better detection of small, overlapping objects, such as tomato clusters, which are critical for precision agriculture.
- Faster Inference: YOLOv8n supports real-time detection with minimal latency, allowing it to process footage.

### Data visualization and management

The ThingsBoard platform offers a web-based interface for data visualization and management. Users can access real-time and historical sensor data presented in dashboards with customizable widgets like charts and graphs. Additionally, captured images with real-time ripening stage detection are displayed, offering a thorough perspective of the greenhouse setting. Users can also configure alerts, generate reports, and manage user access levels within the platform.

Figure 4 above shows an IoT-based farm monitoring system. Sensors (temperature, humidity, light/LDR, soil moisture) connect to an ESP32 microcontroller, which sends data via Wi-Fi to the ThingsBoard cloud platform using the MQTT protocol. A Raspberry Pi with a camera handles image processing, while a farmer’s PC displays the data for analysis. The system enables real-time crop monitoring and precision agriculture.

### Code availability

The custom code used for sensor data acquisition, MQTT-based communication with the cloud, and YOLOv8-based tomato ripeness classification is available at Zenodo: 10.5281/zenodo.16420821^25^. This archived version ensures reproducibility and long-term accessibility. The repository includes Arduino code for the ESP32 microcontroller, Python scripts for model training of YOLOV8 that would run on Raspberry Pi. The code snapshot reflects the version used in the implementation described in this study.

- Sensor data is transmitted via MQTT to ThingsBoard.
- Mmodel training of YOLOV8.

## Hardware implementation: Building the smart greenhouse system

The main challenge of designing the smart greenhouse system was to build low-cost and energy-efficient hardware capable of monitoring, gathering, and controlling physical parameters. The microcontroller unit (MCU) is the heart of the system.

The ESP32 microcontroller was selected due to its built-in Wi-Fi and Bluetooth capabilities, which facilitate wireless communication with the cloud platform. It is programmed using the Arduino IDE to collect sensor data, format it, and transmit it to the cloud. However, one challenge in this implementation is the ESP32’s high energy consumption, particularly when using continuous Wi-Fi transmission. Future iterations may explore power-efficient alternatives such as duty-cycled transmission or LoRa-based communication. The ESP32 operates at 3.3 V input voltage with a maximum CPU current draw of 240 mA, making it suitable for battery-powered greenhouse applications.

**Fig. 5:**
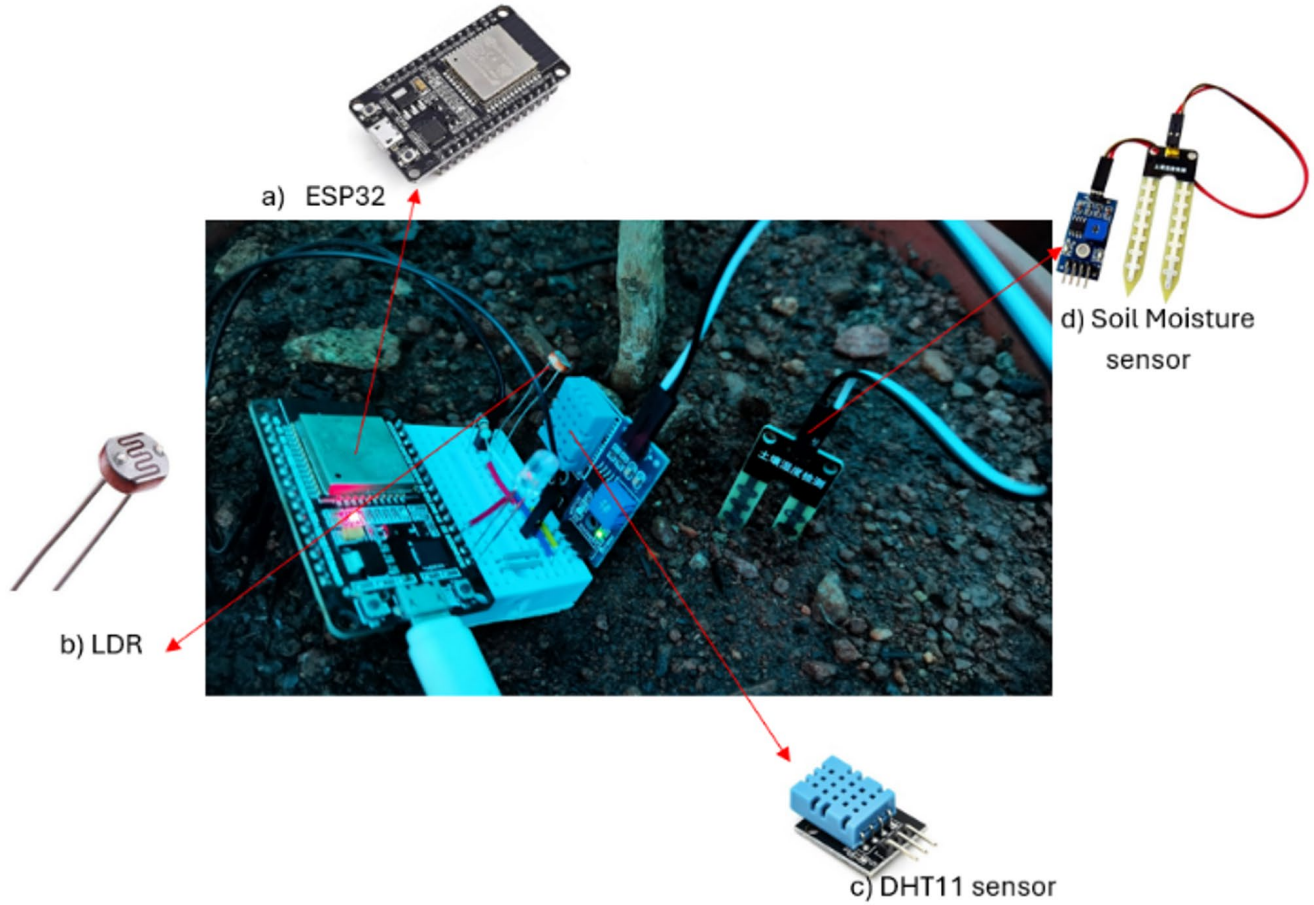
Prototype node.

Figure 5 shows the hardware components of a prototype node, including an ESP32 microcontroller for data processing, an LDR for measuring light intensity, and a DHT11 sensor for monitoring temperature and humidity. These components form the foundation of a sensor node used in smart agriculture systems.

**Fig. 6:**
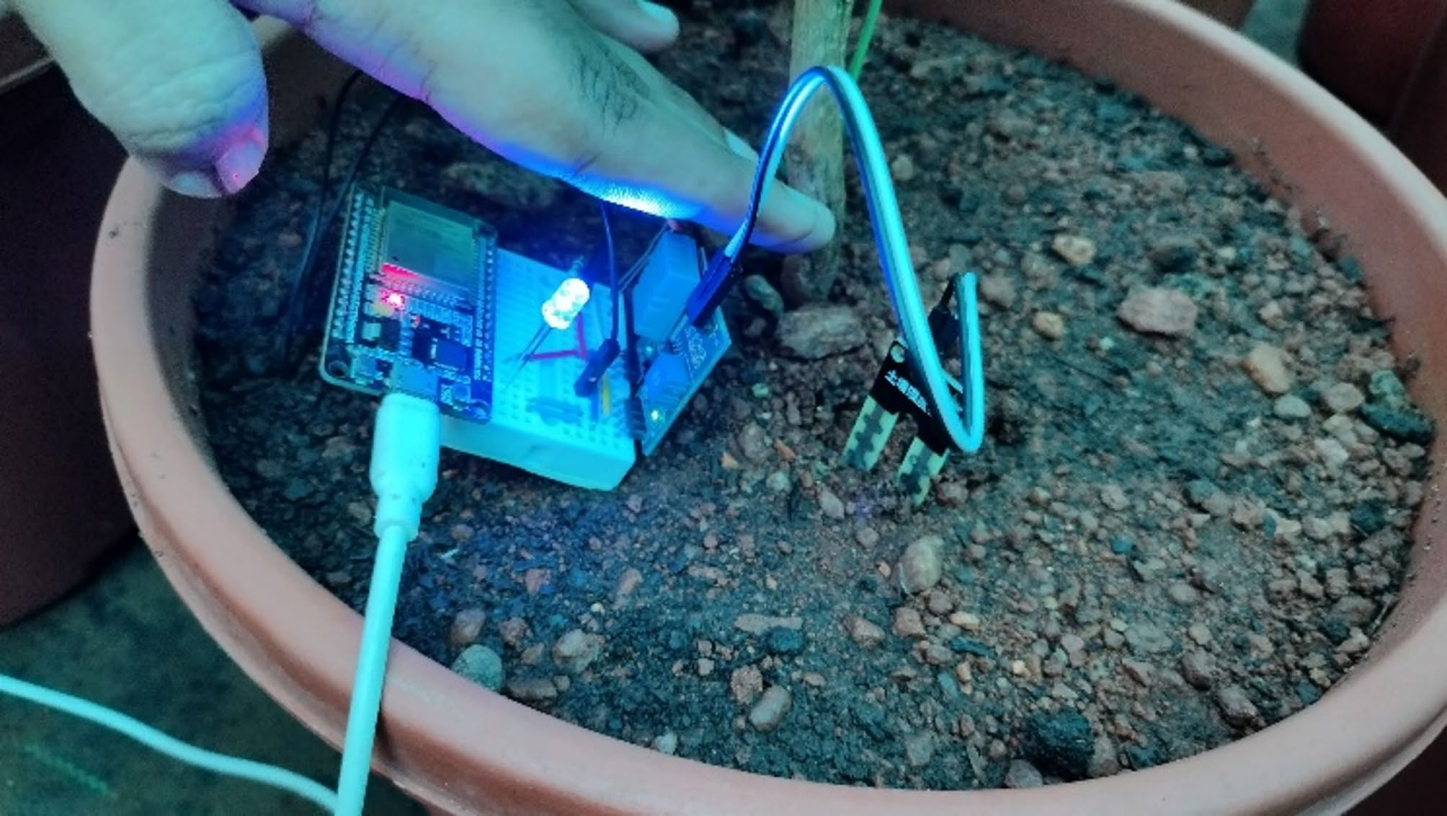
Visualizing LED as the light of greenhouse.

Figure 6 demonstrates the use of LEDs to simulate or supplement natural light within a greenhouse. This visualization highlights the role of artificial lighting in optimizing plant growth conditions, especially in controlled environments where sunlight may be insufficient.

**Fig. 7:**
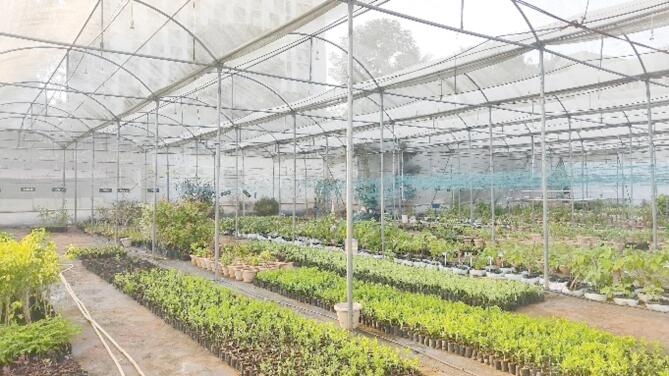
VAIAL’s Greenhouse in VIT, Vellore.

Figure 7 is of the greenhouse located at VIT, Vellore, as part of the VAIAL department. The greenhouse represents an operational setup for studying advanced agricultural techniques to improve crop yields and resource efficiency.

Sensors are crucial for monitoring different environmental parameters in the smart greenhouse system. We used the sensors shown in Table 3.

**Table 3:** Sensors used in the smart greenhouse system.

S No.	Sensors	Model
1.	Temperature and humidity sensor	DHT-11
2.	Light-dependent resistor (LDR)	Generic LDR
3.	Soil moisture sensor	SHT − 10

The soil’s moisture content is measured by the capacitive soil moisture sensor, which is essential for ensuring that tomato plants receive the right amount of watering. The DHT11 sensor monitors the ambient temperature and humidity within the greenhouse, enabling control of ventilation and other climate factors. The LDR detects changes in light intensity, potentially useful for regulating supplemental lighting for the plants.

A Raspberry Pi serves as the image processing unit, enabling real-time monitoring of tomato ripening stages. Unlike prior works that utilized pre-labelled datasets, this study collected images directly from the greenhouse, improving model relevance for real-world applications.

**Fig. 8:**
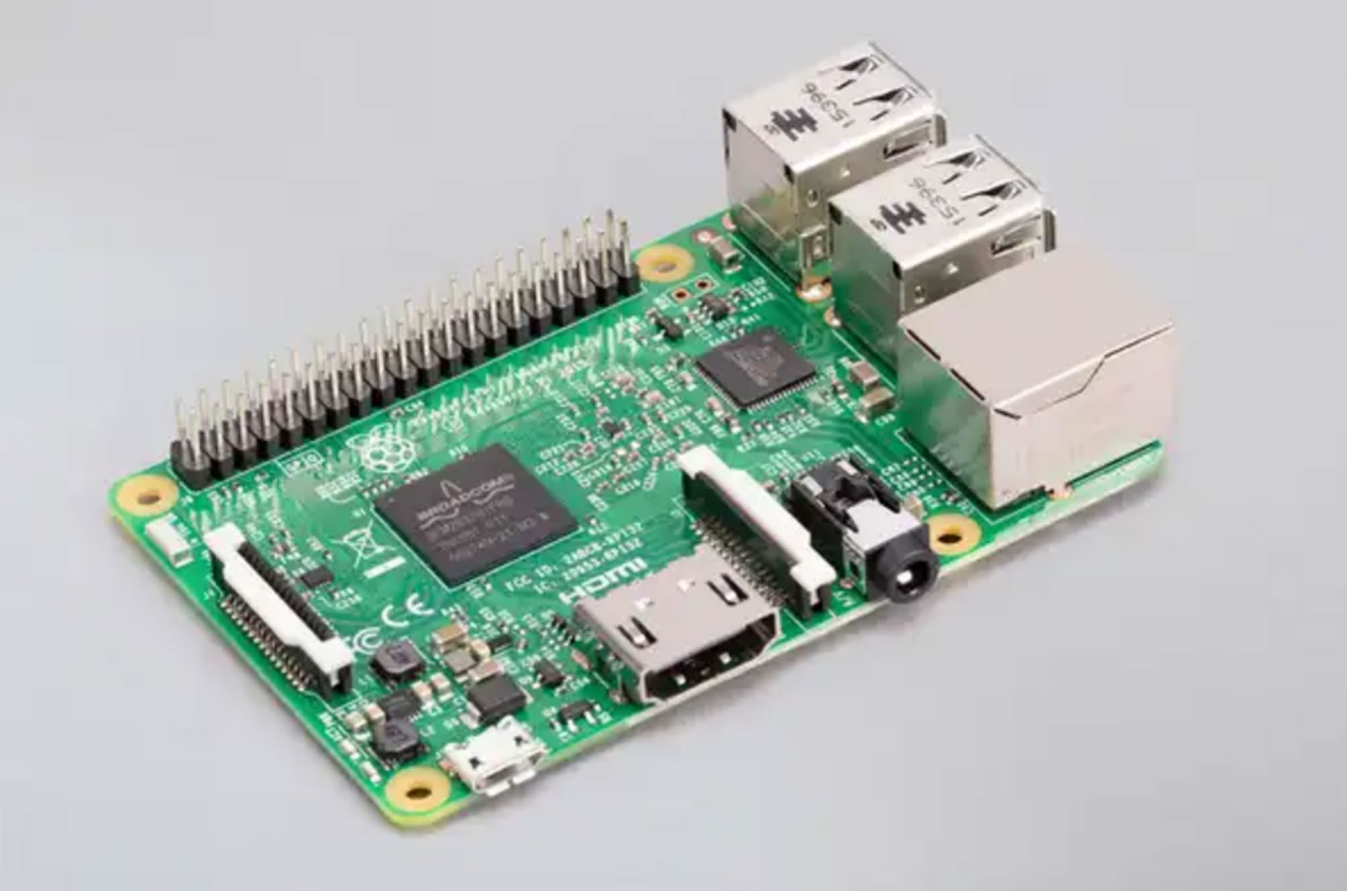
Raspberry Pi.

A Pi Camera attached to the Raspberry Pi makes up the camera module, capturing images of the tomato plants for further analysis, as shown in Fig. 8.

Additional hardware components include jumper wires, a breadboard (optional), and a power supply. Jumper wires are used for connecting the various components, including sensors, and ESP32, as shown in Fig. 8. A breadboard provides a temporary platform for prototyping and testing the hardware connections before finalizing the design. The power supply ensures adequate power delivery to all the components, which could be a USB power bank or a dedicated power supply unit.

The hardware assembly process involves the following steps:

- Connecting the sensors: Following the sensor datasheets and pin configurations, connect the soil moisture sensor, temperature and humidity sensor, and LDR to the ESP32 using jumper wires. ·.
- Set up the Raspberry Pi: Install the Raspberry Pi OS and configure the Raspberry Pi according to its user guide. ·.
- Connect the camera module: Attach the Raspberry Pi camera module following the official instructions.

The single-board computer is a Raspberry Pi, serving as the processing unit for image capture, pre-processing, and running the YOLOv8 model for ripening stage detection. It features a ‘1.2 GHz 64-bit quad-core ARMv8 CPU, an integrated 802.11n wireless LAN and Bluetooth 4.1, Bluetooth Low Energy (BLE), 4 USB ports, a display interface (DSI), and a micro-SD card slot^7^.

## Results: showcasing the platform’s capabilities

**Fig. 9:**
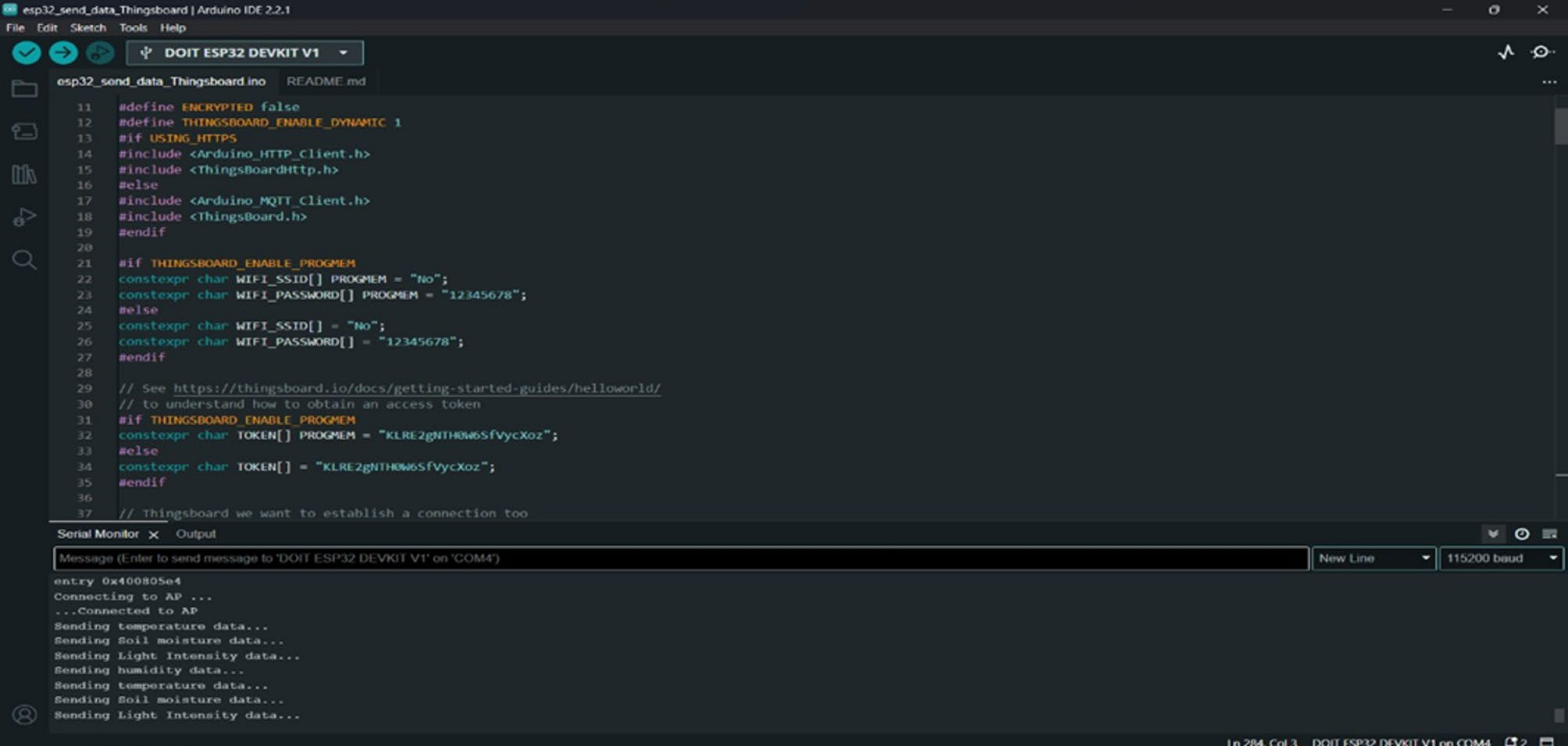
Serial monitor of arduino IDE.

The proposed smart platform was implemented in a controlled greenhouse environment. The sensor network successfully collected real-time data on soil moisture, temperature, and humidity. ESP32 transmitted this data wirelessly to the ThingsBoard platform, where it was visualized on a user-friendly dashboard shown in Fig. 9. The Raspberry Pi captured images of tomatoes at regular intervals. The YOLOv8 model accurately detected tomatoes in the images and classified them according to their ripening stage (green, half-ripened, fully ripened). The processed image data, including bounding boxes and ripening stage labels, was transmitted to the ThingsBoard platform.

### Image with ripening stage detection: Convolution neural network model

**Fig. 10:**
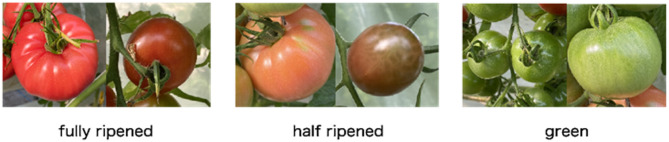
Ripening stages of tomatoes^5^.

**Fig. 11:**
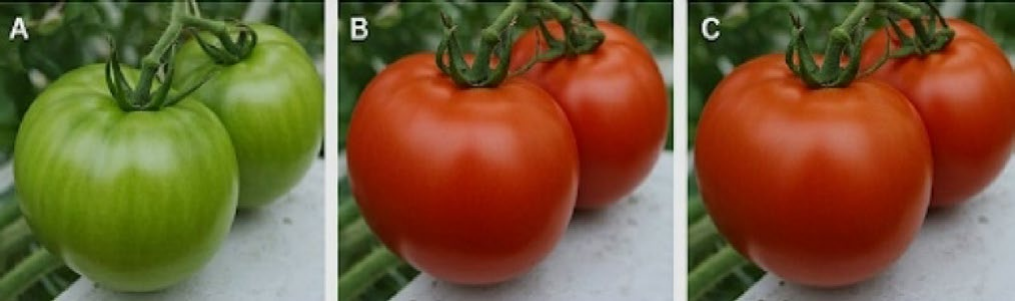
Camera captured images.

**Fig. 12:**
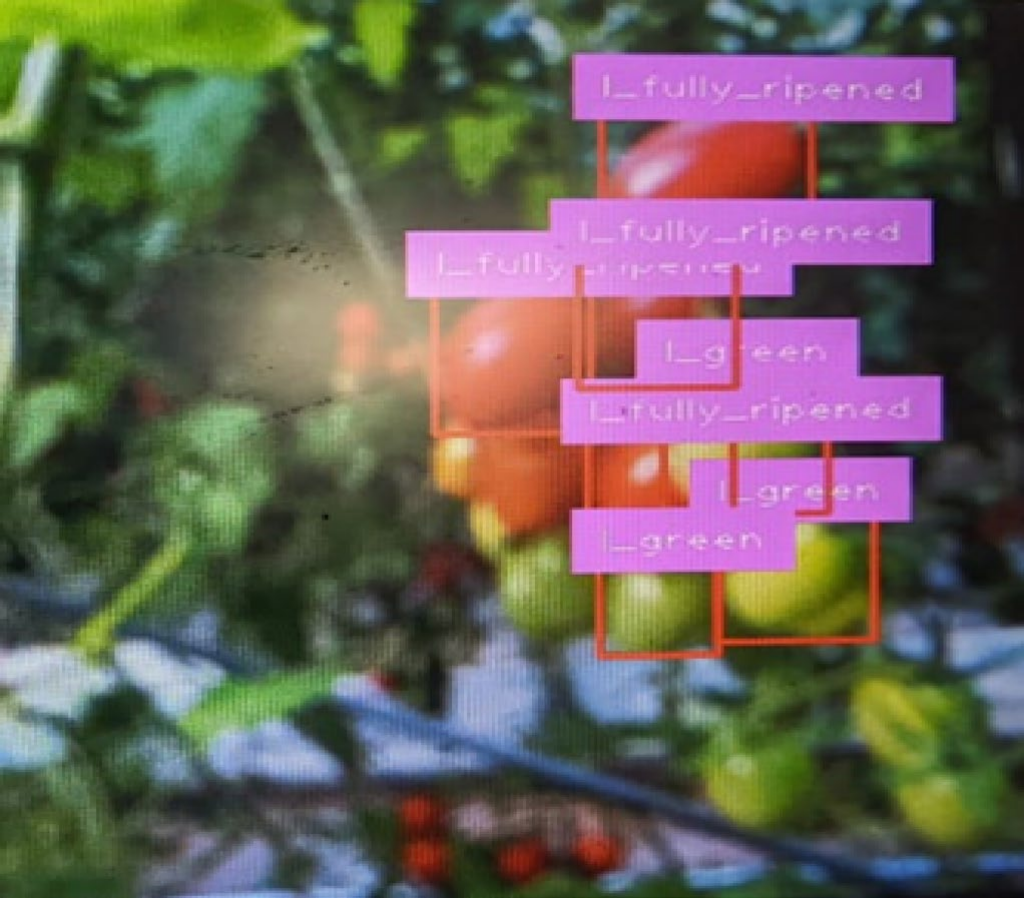
Detection of ripening stage describes bounding box classification.

The YOLOv8 model accurately detected the ripening stages of the tomatoes. The Raspberry Pi captured images of the tomatoes at different stages of ripeness, and the model classified them into green, half-ripened, and fully ripened categories with high accuracy. Figure 10 presents an example image captured by the Raspberry Pi camera. The image overlays bounding boxes around detected tomatoes with labels indicating their respective ripening stages (green, half-ripened, or fully ripened) predicted by the YOLOv8 model. This visual representation provides valuable information for harvest planning and resource allocation shown in Figs. 11 and 12.

### Confusion matrix

To examine the effectiveness of the YOLOv8n model, a confusion matrix was created (Figs. 17, 18, 19). The matrix displays the predicted versus actual classes, allowing us to assess the model’s accuracy for each class. The results show that the model performs well in distinguishing between different ripeness stages. For instance, ‘l_green’ instances are mostly correctly classified, while some misclassifications occur between ‘b_half_ripened’ and ‘b_green’. The confusion matrix reveals that the ‘l_green’ class has the highest number of correct predictions, with a few instances of misclassification in other categories. This indicates that the model can accurately identify green tomatoes, but there is some room for improvement in distinguishing between half-ripened and fully ripened stages.

### Model performance

#### Detection accuracy

The model demonstrates strong object detection capability across all six classes, with the highest accuracy in detecting l_green and l_fully_ripened tomatoes. Detection performance is slightly lower for b_fully_ripened and b_half_ripened, which can be attributed to the lower number of annotated samples.

**Fig. 13:**
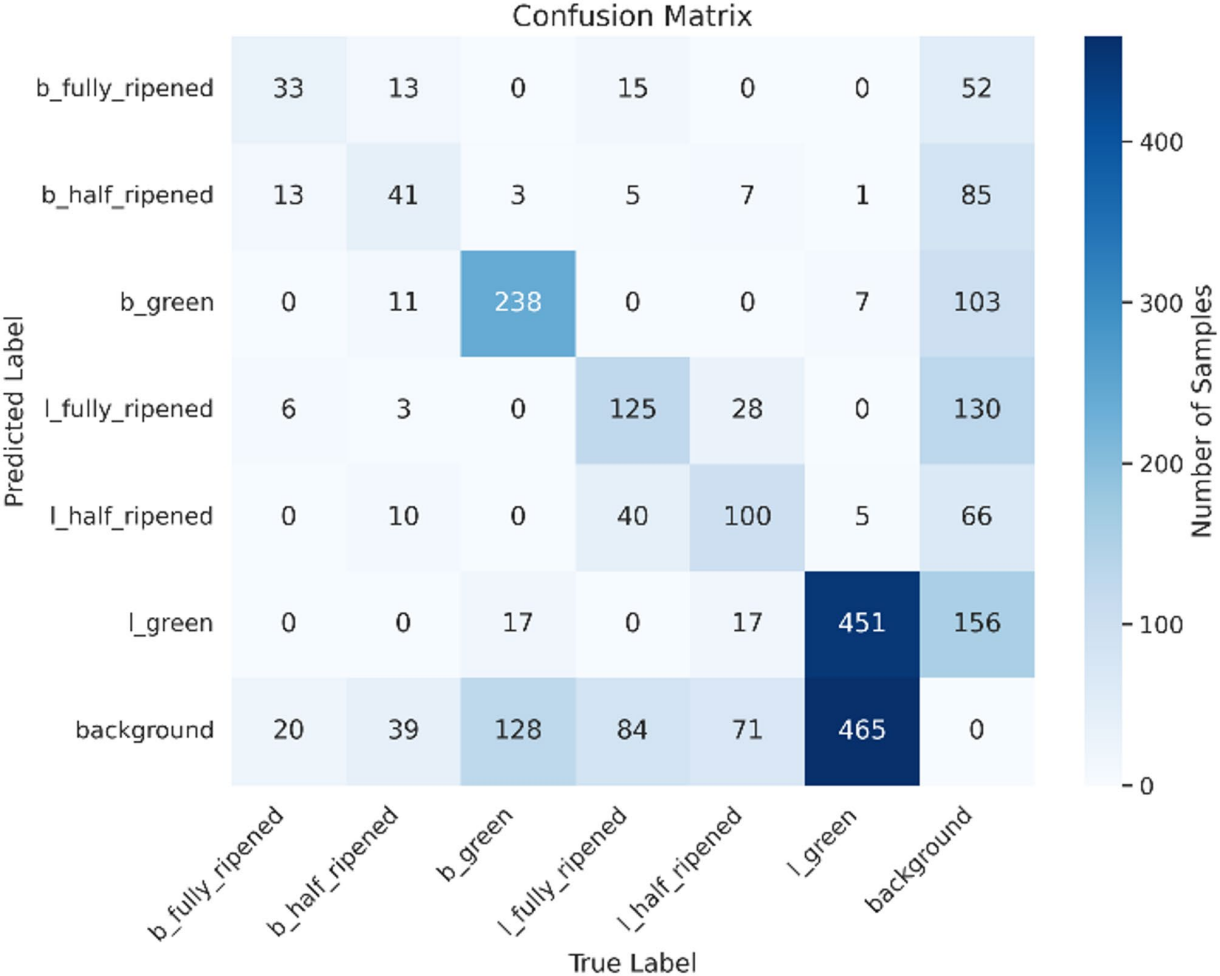
Confusion Matrix of the model.

In Table 4, the precision, recall, f1-score, and support columns show the results. Precision indicates the accuracy of the positive predictions made by the model, recall indicates how well the model can identify the overall positive class, and F1-score indicates the accuracy of the model. Based on the evaluation matrix results from Fig. 13, the model has an accuracy of 52% in testing the test data.

**Table 4:** Parameters for training the model: confusion matrix evaluation results of ripening detection model.

Class	Precision	Recall	F1 Score	Support
b_fully_ripened	0.444	0.344	0.388	93
b_half_ripened	0.362	0.313	0.335	134
b_green	0.625	0.656	0.64	369
l_fully_ripened	0.465	0.433	0.448	289
l_half_ripened	0.453	0.419	0.435	241
l_green	0.489	0.708	0.578	638

#### Visual prediction result

- The output detection images in figure clearly show accurate bounding boxes and class labels overlaid on test images, confirming the model’s robustness under varied lighting and angle conditions.
- Even in dense clusters or partial occlusions, the YOLOv8n model maintained reliable detections, making it viable for real-time agricultural applications.

### Cloud dashboard: visualization and analysis

**Fig. 14:**
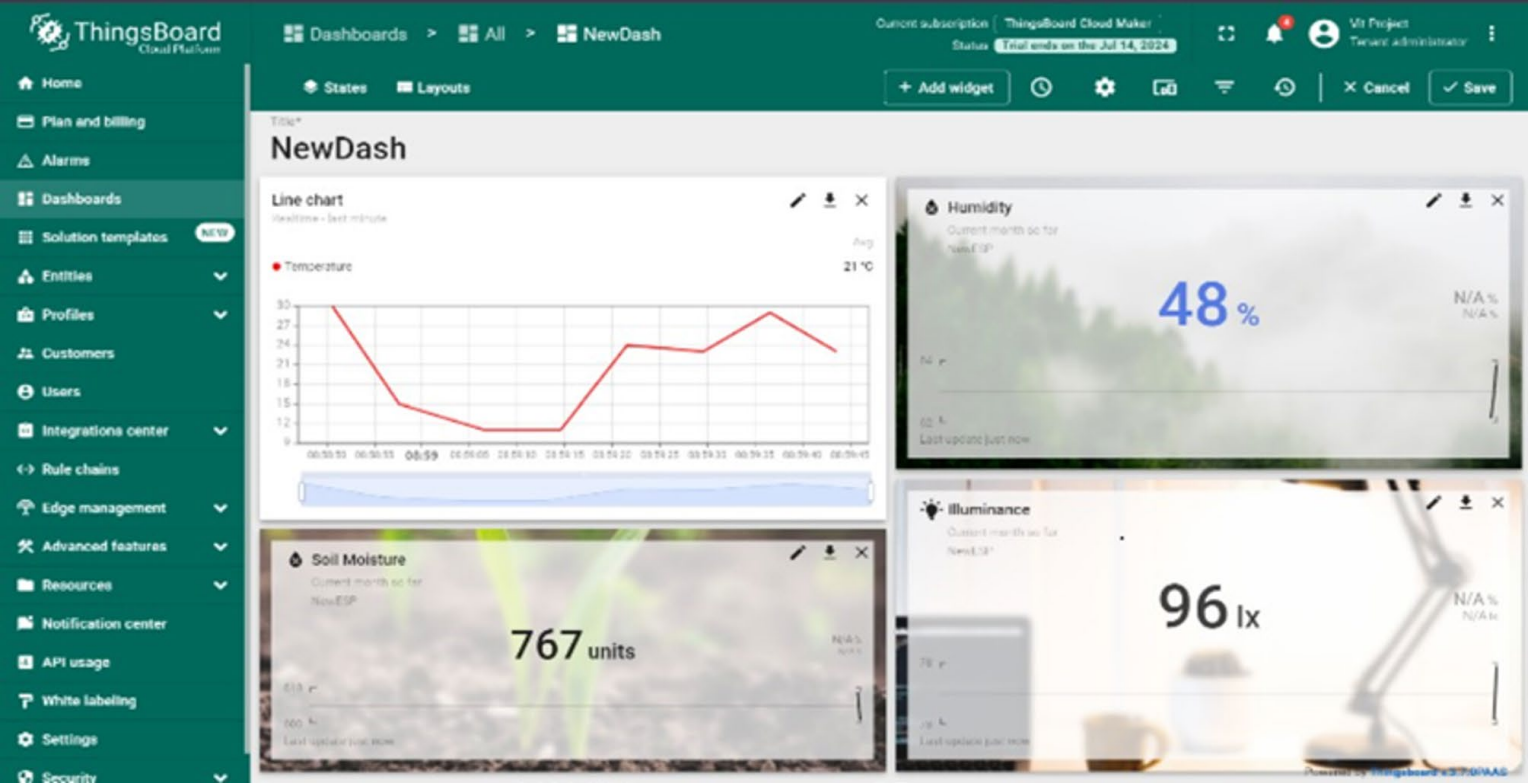
ThingsBoard dashboard.

**Fig. 15:**
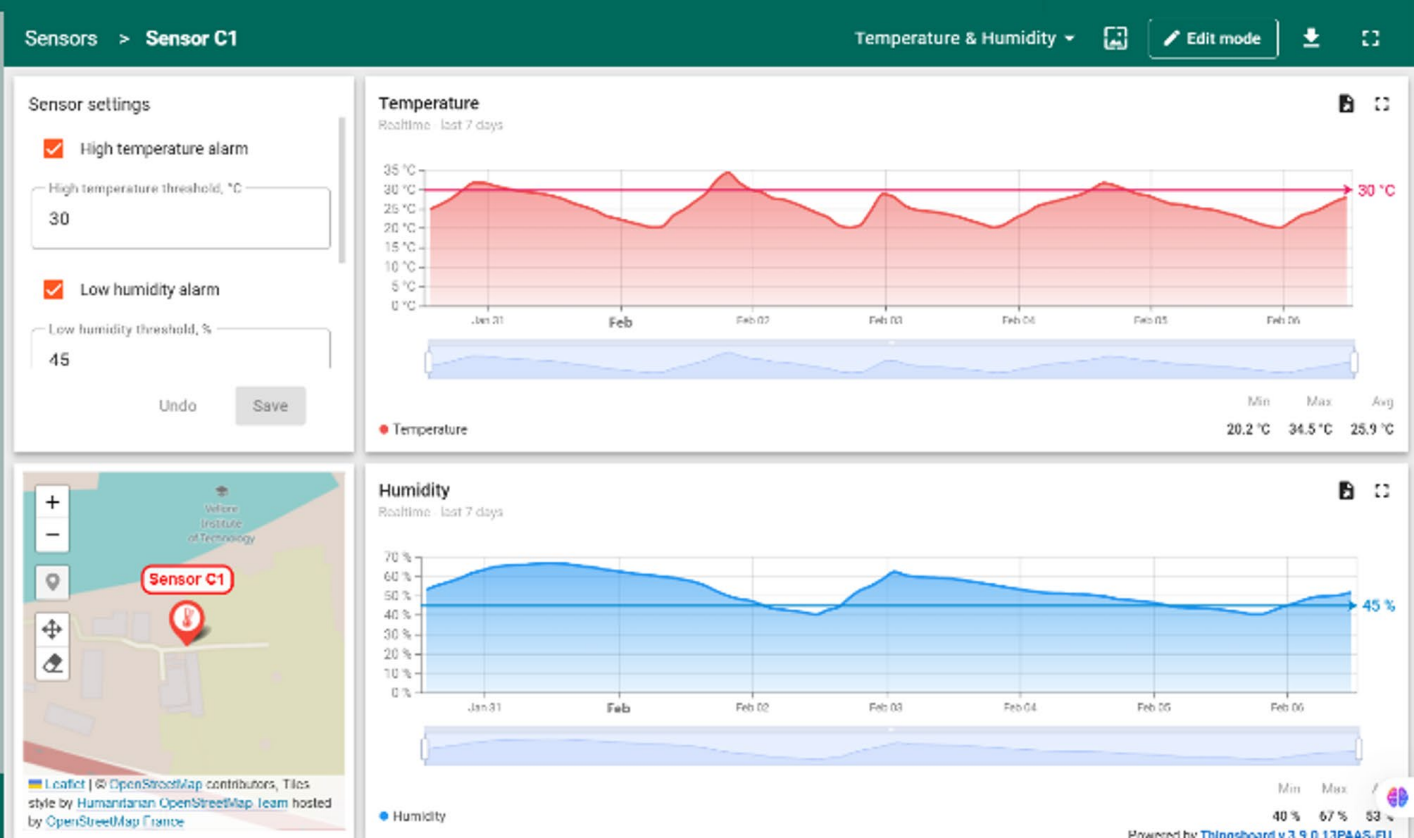
DHT11 data reception.

Figures 14 and 15 depicts the ThingsBoard dashboard displaying real-time sensor data from the greenhouse. The dashboard includes widgets showcasing the current values of soil moisture, temperature, and humidity. Users can customize the dashboard to display historical data in the form of graphs and charts, providing insights into trends and fluctuations over time. Analysing these trends enables farmers to identify potential issues like rising temperatures or declining soil moisture levels and take corrective actions before they negatively impact crop health.

#### Data visualization

**Fig. 16:**
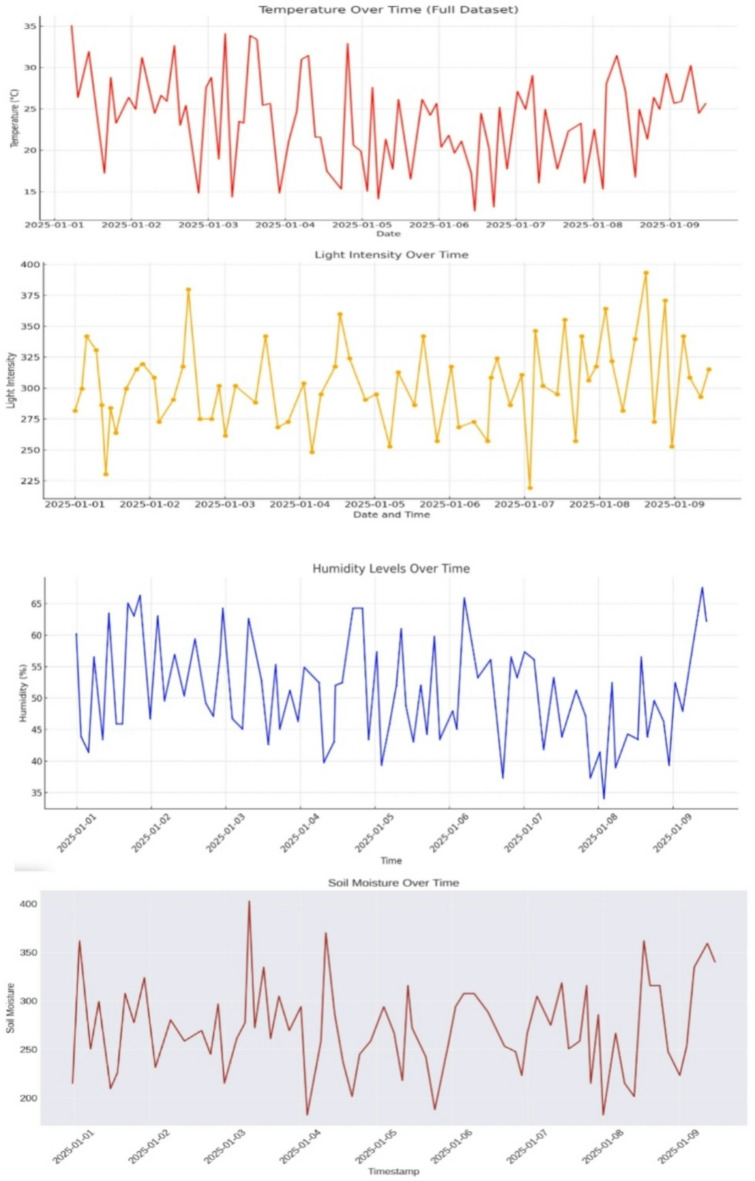
Graphs of data collected by sensors in Thingsboard.

Figure 16 illustrates a sample graph generated from the sensor data collected by the Thingsboard platform. The graph depicts the variations in temperature within the greenhouse over a specific period. The cloud-based dashboard provided a comprehensive view of the sensor data and ripening stages. The dashboard displayed real-time graphs of soil moisture, temperature, and humidity levels, as well as images of the tomatoes with their respective ripening stage.

#### Analysis

In this section, we present the results and analysis of the YOLOv8 model trained to detect the ripening stages of tomatoes. Various visualizations and metrics have been used to evaluate the model’s performance.

**Fig. 17:**
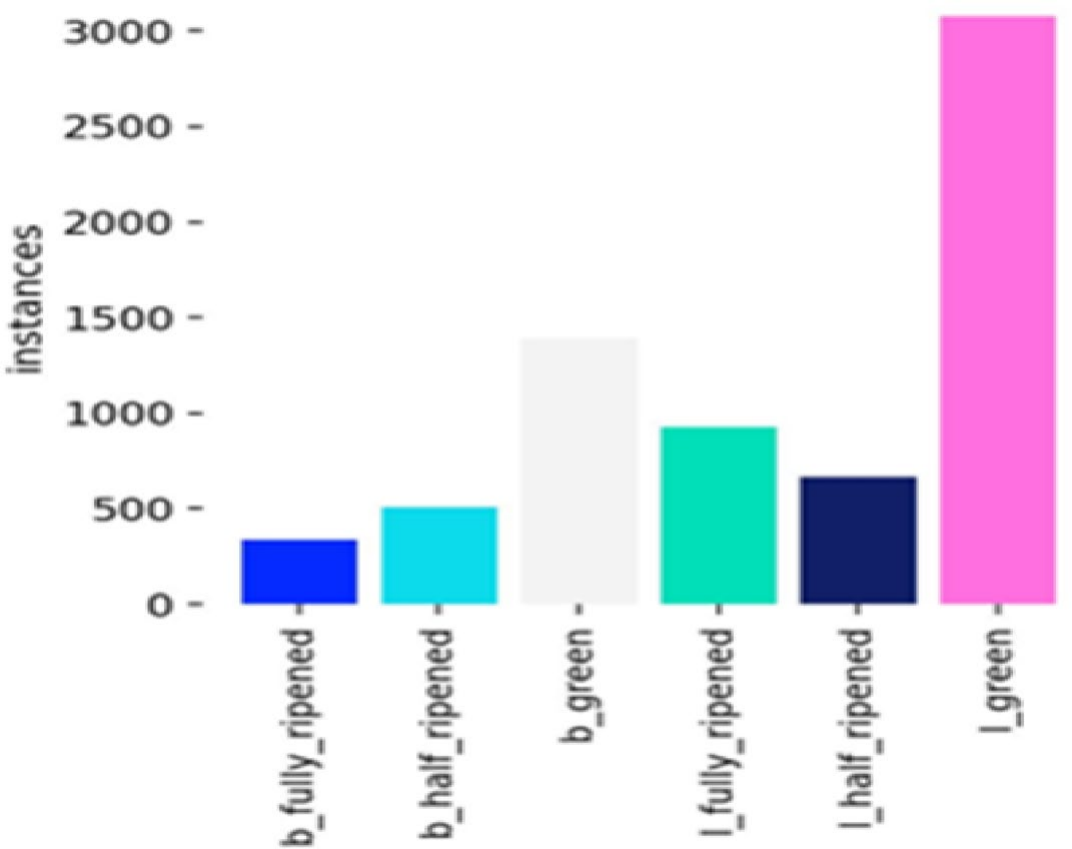
Instance distribution graph.

**Fig. 18:**
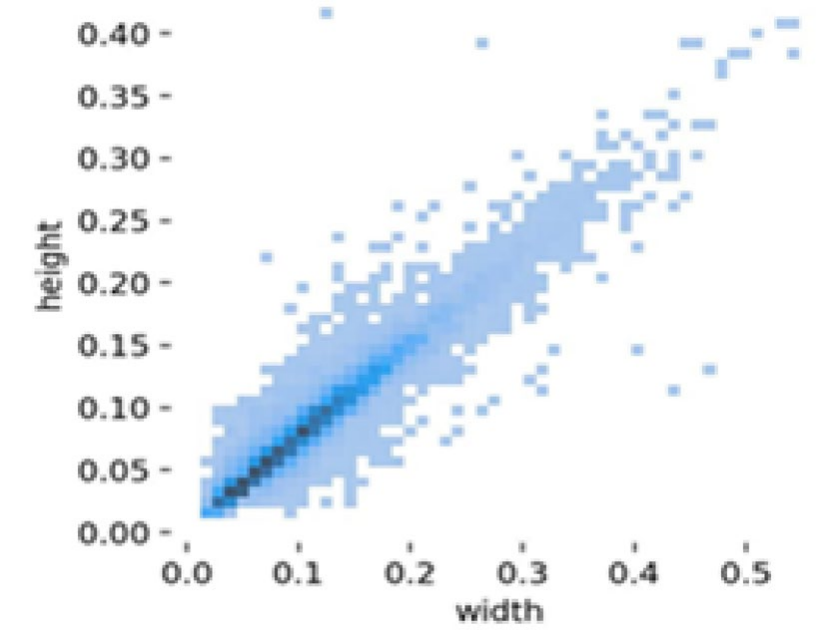
Boundary box distribution.

**Fig. 19:**
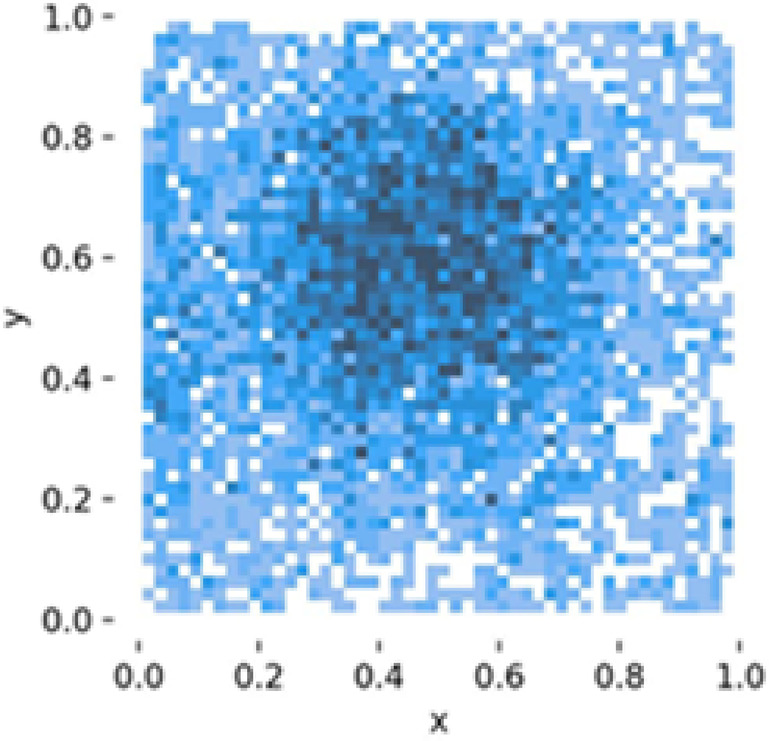
Width and height distributions.

The instance distribution graph (Fig. 17) shows the number of instances for each class (‘b_fully_ripened’, ‘b_half_ripened, b_green’, ‘l_fully_ripened’, ‘l_half_ripened’, ‘l_green’). It highlights that the ‘l_green’ class has the highest number of instances, followed by ‘b_green’. The bounding box distributions (Fig. 18) display the normalized x and y coordinates, showing the density and distribution of bounding boxes in the images. The width and height distributions (Fig. 19) indicate the range of sizes of the bounding boxes, revealing a correlation between width and height.

**Fig. 20:**
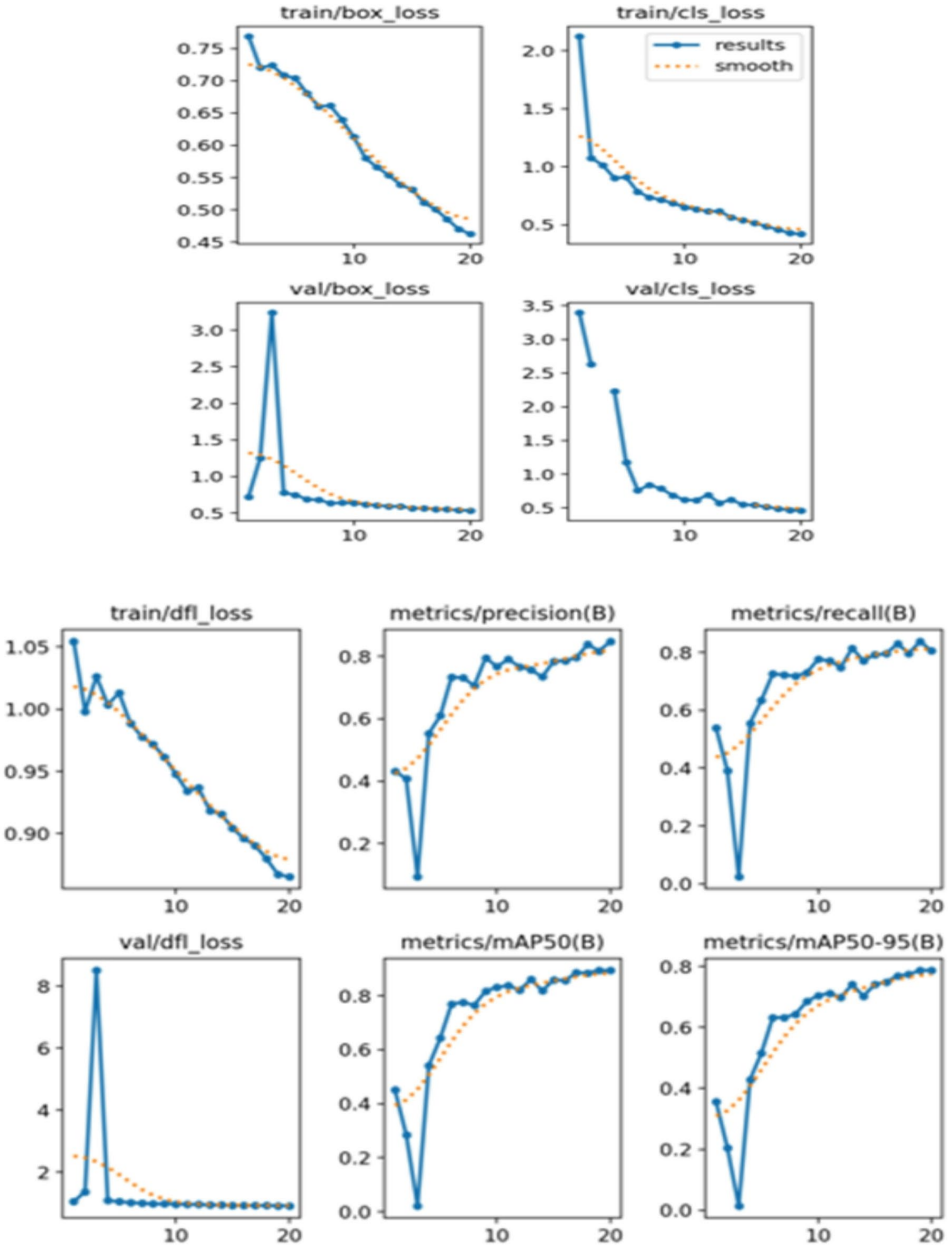
Training and validation loss curves—informative and matches academic standards.

Figure 20 presents the training and validation loss curves over 20 epochs. The training and validation box loss, class loss, and DFL loss are plotted. The results indicate a steady decrease in losses, suggesting that the model is learning effectively. Metrics such as precision, recall, mAP50, and mAP50-95 are also displayed, showing improvements as training progress. These metrics are critical for evaluating the model’s performance in detecting and classifying tomato ripeness stages.

### Energy consumption

Understanding the energy consumption of hardware components is critical for designing efficient and sustainable systems, especially in applications like smart greenhouses where devices are continuously operational. This section presents a detailed analysis of the Raspberry Pi 3 Model B and the ESP32 microcontroller, two essential parts of the smart greenhouse system, and how much energy they require.

#### Energy consumption of ESP32^8^

The ESP32 microcontroller is designed for low-power applications and offers significant energy efficiency. It operates at a voltage of 3.3 V and has distinct power consumption modes depending on its activity:

##### Active mode

When actively processing data or transmitting information, the ESP32 consumes approximately 160 mA.

The power consumption in this mode is calculated as

1 $$\:P\left(active\right)\:=\:V\times\:I\:=\:3.3V\:\times\:\:160mA\:=\:0.528\:W$$

##### Sleep mode

In deep sleep mode, the ESP32 significantly reduces its power consumption to about 10 µA. The power consumption during sleep is:

2 $$\:P\left(sleep\right)=V\times\:I=3.3\:V\times\:10\:\mu\:A=0.033\:W$$

Assuming the ESP32 is active for 12 h a day and in deep sleep for the remaining 12 h, the daily energy consumption is:

3 $$\:Active\:Energy:\:E\left(active\right)=0.528\:W\times\:12\:h=6.336\:Wh$$

4 $$\:Sleep\:Energy:\:E\left(sleep\right)=0.033\:mW\times\:12\:h=0.000396\:Wh$$

##### Total daily energy consumption

5 $$\:E\left(total\right)=6.336\:Wh+0.000396\:Wh=\:\:6.336\:Wh$$

##### Wi-Fi transmission power

Wi-Fi communication is a major factor affecting ESP32’s power usage. The ESP32 uses ~ 260 mA when transmitting data over Wi-Fi, significantly increasing its power draw. To estimate the additional energy consumption:

6 $$\:P(Wi-Fi)\:=\:3.3V\:\times\:\:260mA\:=\:0.858W$$

Wi-Fi Usage Scenario: Assuming the ESP32 transmits data for 15 min per hour throughout a 12-hour active period:

7 $$\:Wi-Fi\:Energy:\:E(Wi-Fi)\:=\:0.858W\:\times\:\:3h\:=\:2.574\:Wh/day$$

Revised Total ESP32 Energy Consumption:

8 $$\:E\left(total\right)\:=\:6.3364\:Wh\:+\:2.574\:Wh\:=\:8.9104\:Wh/day$$

#### Energy consumption of raspberry Pi 3 B^7^

The Raspberry Pi 3 Model B is intended for more demanding computational operations and runs at a higher voltage of 5 V. The way it operates affects how much power it uses:

##### Active mode

When fully operational, the Raspberry Pi consumes approximately 500 mA. The power consumption is:

9 $$\:{P}_{active}=V\times\:I=5\:V\times\:500\:mA=2.5\:W$$

##### Idle mode

When in idle mode, the Raspberry Pi’s consumption drops to about 400 mA. The power consumption is:

10 $$\:{P}_{idle}=V\times\:I=5\:V\times\:400\:mA=2\:W$$

For a continuous operational period of 24 h, the daily energy consumption is:

11 $$\:\text{A}\text{c}\text{t}\text{i}\text{v}\text{e}\:\text{e}\text{n}\text{e}\text{r}\text{g}\text{y}\:{E}_{active}=2.5\:W\times\:24\:h=60\:Wh$$

#### Wi-Fi power consumption

Raspberry Pi Wi-Fi transmission draws ~ 300 mA additional current, leading to increased consumption.

12 $$\:Additional\:power\:due\:to\:Wi-Fi.\:5V\:\times\:\:300mA\:=\:1.5W$$

13 $$\:Estimated\:additional\:energy.\:1.5W\:\times\:\:12h\:=\:18\:Wh/day$$

#### Total energy consumption

14 $$\:E\left(total\right)\:=\:60\:Wh\:+\:18\:Wh\:=\:78\:Wh/day$$

**Fig. 21:**
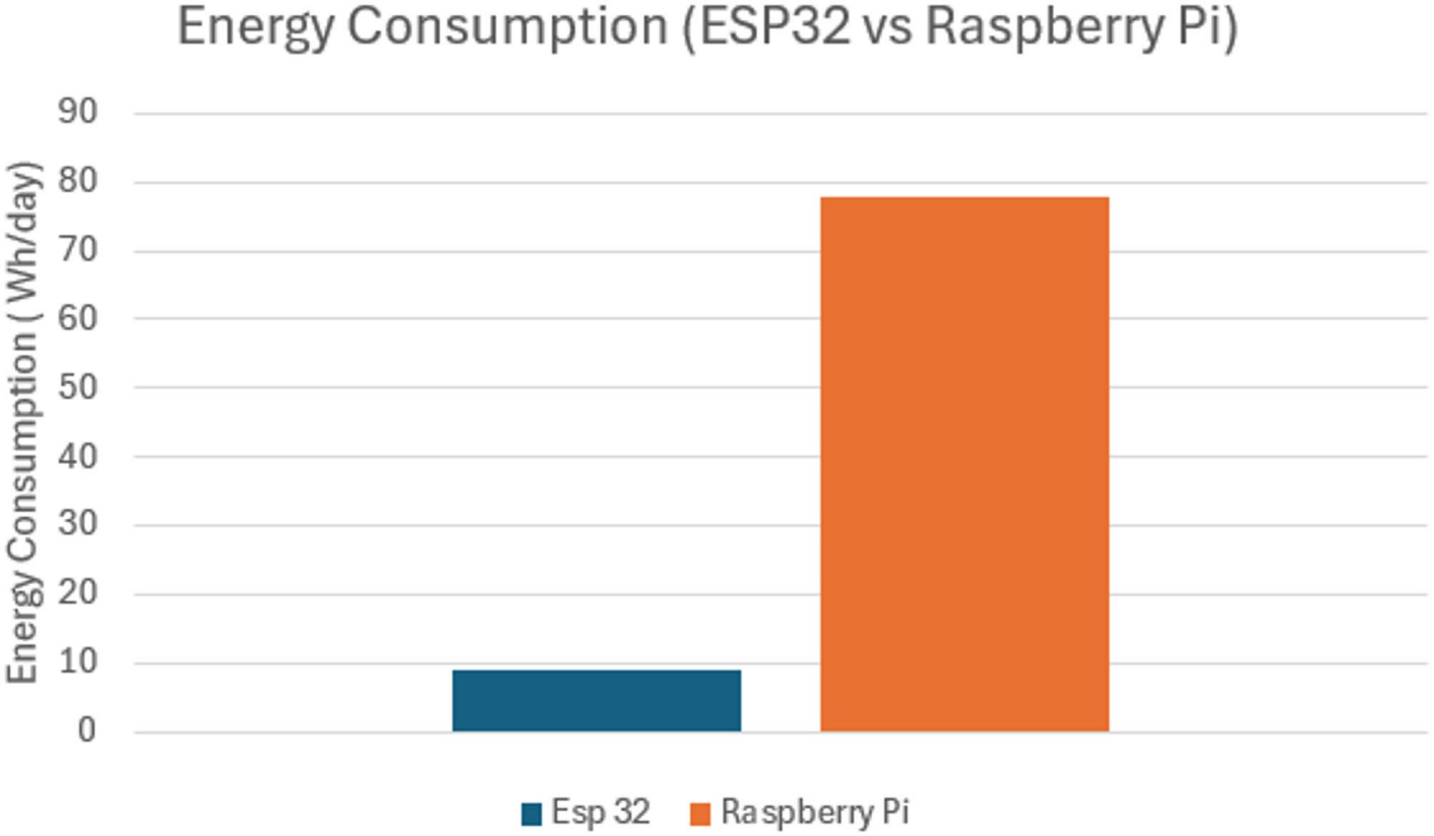
Energy consumption of the devices.

The energy consumption comparison between the ESP32 and the Raspberry Pi reveals substantial differences as shown in Fig. 21.

##### ESP32

Consumes about 8.91 Wh/day when active for 12 h and in sleep mode for 12 h. This low energy requirement highlights its suitability for battery-powered and energy-efficient applications.

##### Raspberry Pi

Consumes approximately 78 Wh/day when continuously active. This higher energy consumption reflects its more intensive computational capabilities and constant operation.

Figure 21 shows the comparative energy usage, highlighting the need for power-efficient alternatives in future deployments (e.g., LoRa, Edge TPU).

The proposed work considered a 9 V Hi-Watt battery as a potential power source. While we have not physically tested this battery, analyzed its theoretical performance based on known specifications and expected power consumption patterns. The Hi-Watt 9 V battery is a commonly available dry-cell battery with an estimated 500mAh capacity, providing approximately 4.5Wh of total energy.

Since the ESP32 operates at 3.3 V, a voltage regulation circuit (such as an LDO or buck converter) would be required to step down the voltage. This introduces additional power losses, reducing the effective energy available to ESP32. Moreover, battery performance is influenced by nonlinear discharge characteristics, meaning that as the battery depletes, its voltage output gradually drops, affecting system stability.

The analysis highlights the importance of selecting a battery based on actual usage requirements rather than theoretical capacity alone. Factors like duty cycle, voltage regulation losses, and environmental conditions can significantly impact battery life. If a longer operational period is required, alternative power solutions such as higher-capacity Li-ion or Li-Po batteries, energy harvesting modules, or solar-powered setups could be considered.

**Fig. 22:**
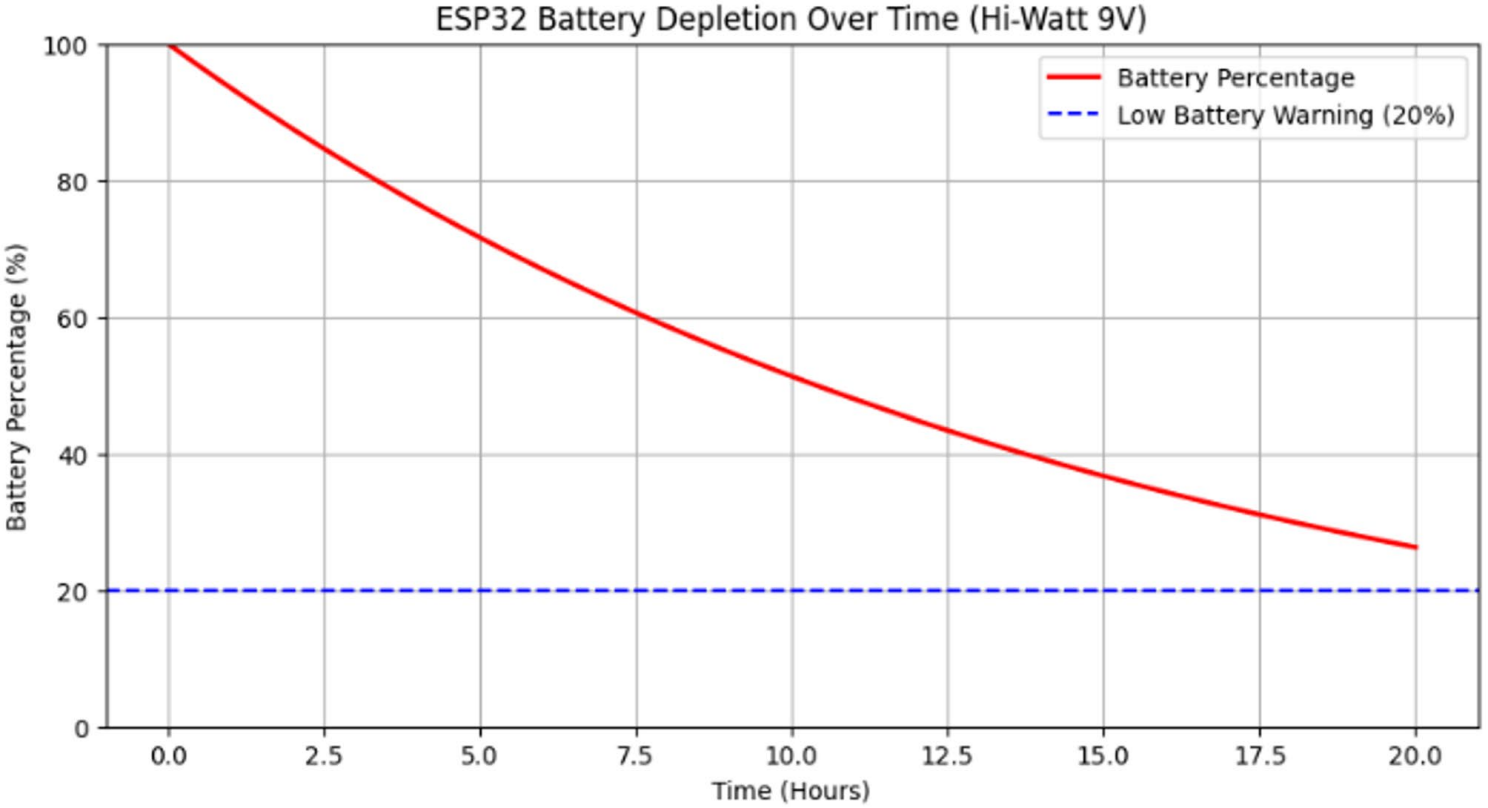
Esp32 battery depletion over time.

The battery depletion curve for the ESP32 shown in Fig. 22 is not linear due to several factors, including internal resistance, chemical reaction kinetics, and discharge rate effects. As seen in the graph, the depletion follows an exponential decay trend rather than a straight-line decline. Initially, energy drains more rapidly due to higher available charge, but as the battery nears depletion, the rate of voltage drop slows down.

The ESP32’s energy consumption varies depending on its operating mode. In active transmission states (Wi-Fi/Bluetooth operations), power draw is significantly higher, whereas in deep sleep modes, the current consumption is minimal. The graph reflects an estimated depletion time of around 17–18 h, assuming a mixed operation profile. Once the battery level reaches the low battery threshold (20%), the system may enter power-saving modes or shut down due to insufficient voltage supply.

### Understanding throughput vs. delay

Throughput refers to the rate at which data is successfully transmitted and received over the network, typically measured in kilobits per second (Kbps) or megabits per second (Mbps). Delay, on the other hand, represents the latency or time taken for data to reach the destination, which includes processing, queuing, and transmission delays.

In an ideal scenario, high throughput and low delay are desirable for efficient data transmission. However, real-world environments introduce factors such as:

- Network Congestion: High data traffic may lead to increased queuing delays.
- Wi-Fi Signal Strength: Weak signal strength can reduce data rates and increase retransmissions.
- Cloud Processing Time: The ThingsBoard platform introduces an additional delay due to server-side data processing and dashboard updates.

**Fig. 23:**
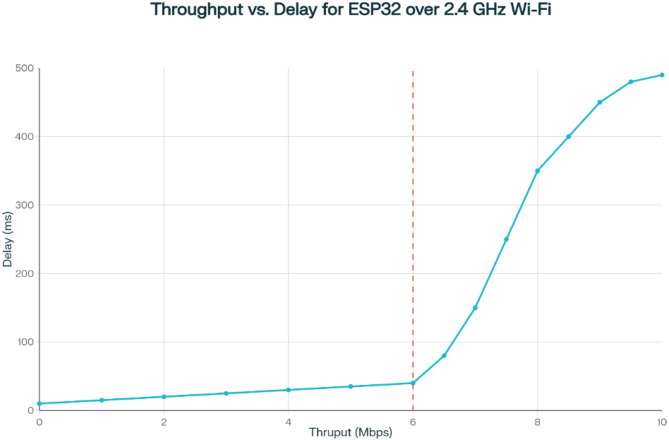
Analysing the throughput vs. delay ESP32 over 2.4 GHz.

From the Throughput vs. Delay graph shown in Fig. 23, it is evident that as throughput increases, delays tend to rise beyond a certain threshold. This can be attributed to limited bandwidth and network contention, where a higher data rate results in increased packet queuing and retransmissions. The ESP32, operating on a typical 2.4 GHz Wi-Fi connection, experiences latencies ranging from a few milliseconds to several hundred milliseconds, depending on environmental interference and cloud response times.

## Discussions and limitations

This smart platform offers a significant leap forward in greenhouse tomato crop monitoring. The integration of IoT and WSN technologies provides real-time data on critical environmental factors like temperature, humidity, and soil moisture. This empowers farmers to make informed decisions for optimized growth conditions, potentially leading to increased yields and improved crop quality. Furthermore, the incorporation of image processing and deep learning through the YOLOv8 model offers a valuable tool for monitoring tomato ripening stages. By accurately identifying ripening tomatoes, farmers can plan harvests more effectively, reducing the risk of overripe or underripe fruit reaching consumers. However, there are limitations to consider,

- Model Performance in Greenhouse Environments: The YOLOv8n model achieved a classification accuracy of 52.8% on the tomato ripeness detection task, which is considered modest for deployment in real-world agricultural settings. This outcome is primarily due to the limited size and diversity of the dataset, which consisted of only 804 annotated images collected under varied lighting and occlusion conditions. Improving accuracy will require curating a significantly larger and more balanced dataset that represents all ripeness classes across different environmental scenarios. Additionally, the model can benefit from fine-tuning through advanced training techniques such as data augmentation, transfer learning with deeper architectures, and hyperparameter optimization. These measures will help improve generalization and robustness, ultimately enhancing performance in operational conditions.
- Scalability and Adoptability - The proposed smart IoT agriculture system is scalable and easy to adopt for real-world use. Its modular design with ESP32 and Raspberry Pi allows adding more sensors and edge devices to support farms of different sizes. MQTT and cloud platforms like ThingsBoard enable efficient data management and smooth scaling. The system adapts to various crops through customizable sensors and retrainable deep learning models. Edge computing supports real-time processing, reducing cloud reliance and saving energy. Low-cost components, user-friendly dashboards, and remote monitoring make deployment simple for farmers. Its flexibility allows localization to different farming needs, promoting practical and sustainable precision agriculture.
- Reliance on Internet Connectivity: The platform depends on a reliable internet connection for seamless data transmission to the cloud platform. Disruptions or outages could hinder real-time monitoring and potentially delay responses to critical changes in the greenhouse environment. Future work will explore edge processing techniques to reduce cloud dependency and ensure local decision-making during network downtimes.
- Energy Consumption and IoT Protocol Selection: The ESP32’s continuous Wi-Fi transmission contributes to high power usage, reducing operational efficiency for battery-powered setups. Future optimizations include duty-cycled operation, alternative communication protocols such as LoRa and ZigBee, and power management techniques like dynamic frequency scaling to reduce energy consumption.
- Future enhancements can explore the integration of hardware accelerators such as the Edge TPU, TensorRT, or NVIDIA Jetson Nano to support local decision-making during internet outages. These devices are designed to run deep learning models efficiently on the edge, enabling faster and more reliable inference without relying on continuous cloud access. Incorporating such accelerators would improve the system’s ability to process image data in real-time, maintain autonomous operations like ripeness detection and actuator control, and ensure uninterrupted performance in remote or connectivity-constrained environments.
- Limited Deployment Scope: The study tested only one prototype node, limiting the ability to validate system scalability. Future work will involve multi-node deployment to improve spatial coverage and ensure a more comprehensive monitoring system.
- Real-Time Processing Constraints: The YOLOv8 model runs on a Raspberry Pi, but inference speed is limited, leading to potential delays in processing image data. Optimizations such as model pruning, quantization, and integration with specialized accelerators (e.g., Edge TPU, NVIDIA Jetson Nano) will be explored.
- Actuator Control Logic: The current system lacks full automation for controlling actuators such as irrigation and ventilation. Future improvements should ensure that sensor-based actuations (e.g., heating, irrigation) are triggered based on multiple sensor inputs rather than isolated measurements, preventing incorrect responses to environmental changes.

## Conclusion

This article presents a novel smart platform for monitoring tomato crops in greenhouses. The platform capitalizes on the strengths of Internet of Things (IoT) and Wireless Sensor Networks (WSNs) to establish a real-time data collection system for crucial environmental factors impacting tomato growth. By integrating image processing and a YOLOv8 deep learning model, the platform offers the ability to automatically detect tomato ripening stages. This empowers farmers with real-time insights for informed decision-making, potentially leading to optimized crop growth, improved yield, and minimized harvest losses. However, limitations exist. The accuracy of the YOLOv8 model is contingent upon the quality of training data, and its performance might be susceptible to variations in lighting conditions, camera angles, and occlusions within the greenhouse environment.

The YOLOv8 model demonstrated promising performance in detecting and classifying the ripening stages of tomatoes. Quantitatively, the model achieved a mean Average Precision (mAP) of 52.8% at an average recall of 0.478, based on nano model of YOLOv8 for such less number of images in the dataset and an epoch of 25, this is a significant number for initial training and can be improved in future working. The YOLOv8 model demonstrated high accuracy in detecting and classifying the ripeness stages of tomatoes. The instance distribution and bounding box analysis provided insights into the dataset’s characteristics. The training and validation loss curves indicated effective learning, with decreasing loss values over epochs. The confusion matrix further validated the model’s performance, showing high accuracy for most classes.

Additionally, the platform’s effectiveness relies on a dependable internet connection for seamless data transmission to the cloud platform. Future research will explore integrating edge computing solutions to enhance offline processing capabilities. Despite these limitations, the proposed platform presents a significant advancement in greenhouse tomato crop monitoring. Future work can focus on enhancing the robustness of the YOLOv8 model for diverse environmental conditions and incorporating additional sensors (e.g., CO₂, pH, and nutrient monitoring) to create a more comprehensive data set. This will pave the way for the development of even more sophisticated models capable of not only identifying ripe tomatoes but also predicting potential issues and optimizing greenhouse conditions for superior crop management and yield. Machine learning-based predictive models for disease detection and climate forecasting will also be explored. Overall, this smart platform offers a promising approach for sustainable, data-driven, and automated tomato production in greenhouses.

## Data Availability

The data that support the findings of this study are available from the corresponding author upon reasonable request.
